# Genetic screening identifies WNT5A/RYK as a determinant of extracellular vesicle fate

**DOI:** 10.1126/sciadv.aeb2877

**Published:** 2026-07-31

**Authors:** Julia Dancourt, Satish Babu Moparthi, Jeanne Lainé, Shéryl Bui, Jianhang Wang, Maud Chevé, Yingke Xu, Stéphane Vassilopoulos, Grégory Lavieu

**Affiliations:** ^1^ INSERM U1334, CNRS UMR8175, Université Paris Cité, NABI, UFR Sciences Fondamentales et Biomédicales, 45 rue des saint Pères, Paris, France.; ^2^ Sorbonne Université, INSERM, Institute of Myology, Centre of Research in Myology, UMRS 974, Paris, France.; ^3^ Department of Biomedical Engineering, MOE Key Laboratory of Biomedical Engineering, State Key Laboratory of Extreme Photonics and Instrumentation, Zhejiang Provincial Key Laboratory of Intelligent Sensing and Advanced Medical Instrument, Zhejiang University, Hangzhou, Zhejiang 310027, China.

## Abstract

Extracellular vesicles (EVs) are natural mediators of intercellular communication through the transfer of cytoplasmic material from donor to recipient cells. The molecular mechanisms governing EV uptake and cargo delivery in recipient cells remain largely elusive. To address this gap, we performed a genetic screening interrogating 300 genes of the dynamic surfaceome for their involvement in EV uptake. Activators found in the screening establish a previously unknown connection between EV uptake and Wnt pathways. We then observed that, unlike WNT-naïve EVs that are primarily trafficked to lysosomes in recipient cells, WNT5A-bearing EVs linger in early endocytic structures. This rerouting is dependent on the expression of RYK, an established co-receptor for WNT5A. Our results depict a mechanism underlying EV functional heterogeneity as well as reveal a potential regulation system for noncanonical WNT signaling.

## Teaser

WNT5A-decorated EVs follow a RYK-specific endocytic route.

## INTRODUCTION

Once dismissed as mere cellular debris, extracellular vesicles (EVs) are now recognized as key mediators of intercellular communication, facilitating the transfer of proteins, lipids, metabolites, and nucleic acids between donor and acceptor cells (*1*, *2*). EVs in the secretome are highly heterogeneous in their subcellular origin, size, and cargo content but reflect the physiological state of the donor cell at a given moment (*3*). Consequently, EVs derived from different cell types or conditions—such as stress, differentiation, or disease—exhibit distinct compositions and signaling properties (*4*). While progress has been made in understanding EV biogenesis, the molecular mechanisms governing their uptake by acceptor cells remain largely unresolved.

Likely due to their heterogeneous nature, EV uptake routes appear highly context specific, with no universal principle governing their internalization (*5*). While several ligand-receptor interactions have been proposed to direct EVs to specific recipient cells (*6*, *7*), EV tropism is generally considered broad (*8*, *9*). A recent study showed that EV uptake is nonsaturable and thus does not seem to require a specific receptor on the acceptor cell membrane (*10*). Additionally, quantitative analyses indicate that EVs are primarily internalized through fluid-phase endocytosis (macropinocytosis), with cargo delivery occurring upon endosomal acidification (*11*, *12*). However, while these mechanisms have been observed at the cellular level, the molecular machinery governing EV internalization and content release remains unidentified. This raises several key questions: Do specific EV types favor particular endocytic routes? How does EV cargo escape from endosomes into the cytoplasm?

While EVs deliver a variety of molecules into the acceptor cell cytoplasm, they can also mediate plasma membrane signaling, including morphogen signaling (*13*). Certain morphogens, such as WNT (wingless-related integration site) and Hedgehog, are highly hydrophobic and thus secreted on EVs (*14*, *15*). Although WNT signaling is best known for its role in embryogenesis, its dysregulation has been implicated in diseases such as cancer (*16*). The WNT family consists of 19 members in humans, which activate signaling through interactions with specific receptors and co-receptors. WNT signaling is broadly classified into two pathways: canonical and noncanonical, each engaging distinct WNT-receptor pairs (*17*, *18*). Canonical signaling, mediated by WNT1-class ligands (e.g., WNT2, WNT3, WNT3A, and WNT8A), involves binding to a Frizzled (FZD) receptor, 1 of 10 in humans, and the LRP5/6 co-receptor. This interaction inhibits glycogen synthase kinase 3 (GSK3), stabilizes β-catenin, and promotes its nuclear translocation to activate WNT target gene transcription (*19*). In contrast, noncanonical WNT signaling, which is β-catenin independent (*20*), is driven by WNT5A-class ligands (e.g., WNT4, WNT5A/B, WNT6, WNT7, and WNT11) binding to FZD receptors and alternative co-receptors such as RYK or RORs. This activates two main pathways: the planar cell polarity pathway and the calcium-dependent pathway (*21*). While the canonical pathway primarily regulates cell fate decisions such as proliferation and survival, the noncanonical pathway governs differentiation, cell polarity, and migration. Although EVs have recently been described as natural WNT carriers (*14*, *22*, *23*), the fate of WNT-bearing EVs, particularly their interactions with specific receptor–co-receptor complexes, remains poorly understood.

In this study, we aimed at elucidating the molecular mechanisms regulating EV uptake by conducting an arrayed genetic screening targeting 300 genes encoding transmembrane proteins localized at the plasma membrane or within the endolysosomal compartment of HeLa cells. We designated this group of genes as part of the “dynamic surfaceome.” Our analysis identified several WNT pathway regulators among EV uptake activators. We then independently tested and demonstrated that Wnt5A-bearing EVs followed a distinct uptake route compared to WNT-naïve or Wnt3A-bearing EVs and that this rerouting was signaling independent. Instead, we found that Wnt5A-EV binding and internalization was influenced by the expression of the WNT5A co-receptor RYK, indicating that co-receptor availability dictates WNT5A-EV dynamics. Unlike WNT-naïve EVs that traffic through the endolysosomal pathway, Wnt5A-EVs remained associated with early endosomes, leading to enhanced cargo delivery into recipient cells. Our findings provide new insights into EV uptake mechanisms and, more specifically, the biology of WNT-bearing EVs.

## RESULTS

To identify new genes involved in EV uptake, we decided to perform a genetic screening on EV recipient cells. The CRISPR-Cas9 genome editing system has proven to be the tool of choice for genetic screenings as it is highly specific, efficient, and amenable to high-throughput functional genomics (*24*). Given that our EV uptake assay (*10*) was not based on single-cell analysis and not directly amenable to negative selection, we decided to perform the screening in an arrayed (plate-based) format on a set of 300 preselected targets.

We custom-designed a single guide RNA (sgRNA) bank targeting genes encoding proteins that we chose as being part of HeLa cells’ dynamic surfaceome based on three criteria. (i) Human Protein Atlas (*25*): We selected proteins annotated in the “plasma membrane” and “vesicles” datasets, retaining only single-pass transmembrane proteins to prevent overrepresentation of G protein–coupled receptors (GPCRs). (ii) SURFY surfaceome predictor (*26*): We filtered the selection using the SURFY machine learning–based surfaceome predictor to ensure relevance to HeLa cells. (iii)

**Fig. 1.**
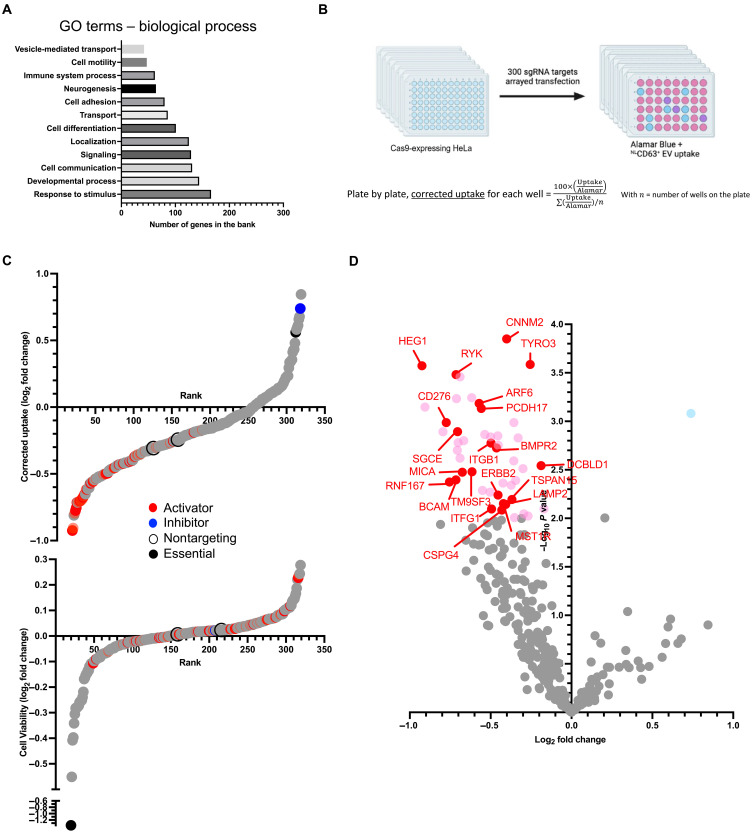
Genetic screening for EV uptake on the dynamic surfaceome. (**A**) Gene Ontology (GO) term enrichment analysis (https://geneontology.org/) was performed on the genes targeted by the dynamic surfaceome sgRNA bank. (**B**) Screening workflow: The 300 genes of the sgRNA bank were individually knocked out in HeLa^CRISPR^ cells before incubation with luminescent NLCD63-EVs and multiplexed cell viability assessment with alamarBlue. The calculation of the “corrected EV uptake” parameter is indicated. (**C**) Genes were ranked by effect on corrected EV uptake (top) or cell viability (bottom). The positions of activators and inhibitor [same cutoff as in (D)] are color coded. Nontargeting sgRNAs are represented. One gene was found to be essential in the cell line tested (LRIG2) as its knockdown resulted in less than 40% cell viability. (**D**) Volcano plot depicting screening results. The log_2_ fold change of corrected EV uptake values were plotted against the −log_10_ of the *P* value for each gene tested. Of all the 48 activators (only cutoff, *P* < 0.01) depicted in pink, 20 of them (red) are involved in Wnt pathways, either through published evidence or through BioGRID interaction networks (see extended data 1).

Quantitative Organellar Proteomics (*27*): We excluded poorly expressed proteins based on proteomics profiling. To refine the gene list, we minimized redundancy within protein families and included a small number (<10%) of additional genes of interest, such as ARF6, a known macropinocytosis regulator, as a positive control (extended data 1). Figure 1A confirms that most targeted genes are involved in cell communication, adhesion, or transport.

We next developed a Cas9-expressing acceptor cell line (HeLa^CRISPR^, fig. S1A) with efficient genome-editing capabilities (fig. S1, B and C). Preliminary experiments validated HeLa^CRISPR^ as a suitable model for conducting an arrayed CRISPR-Cas9 screen (fig. S1, D and E). We then optimized the parameters for our high-content EV uptake bioassay. First, we isolated and characterized luminescent EVs from HeLa^NLCD63^ cells (fig. S1, F and G) that expressed NanoLuciferase (NL)–tagged CD63, an established EV reporter (*10*, *28*), showing an average size of 151.8 nm, enrichment in canonical EV markers (CD63 and Alix), and depletion of cellular markers (calnexin). The NLCD63-containing EV uptake bioassay has been described previously (*10*, *11*), and, for the screening, we opted for short uptake kinetics to minimize lysosomal degradation, which could be a confounding factor if influenced by genes in our sgRNA library. After 24 hours, most internalized EVs were trafficked to lysosomes, as evidenced by their stabilization with E64d, a cysteine protease inhibitor (fig. S1H). To circumvent this, we set the EV incubation period to 4 hours for the screening. Additionally, to control for potential gene knockout toxicity, we (i) verified that EV uptake remained linear across a wide range of cell confluencies (fig. S1I) and (ii) multiplexed the EV uptake assay with a cell viability assay, using viability as a corrective factor to refine our screening parameter (Fig. 1B).

Several EV uptake activators were identified using criteria that accounted for both effect size and statistical significance along with a single prominent inhibitor that is under separated investigation (Fig. 1, C and D). As expected, there appeared to be no correlation between EV uptake modulation and cell viability (Fig. 1C). Moreover, the top hit, HEG1, was confirmed as an activator in a secondary test (fig. S1J), validating our approach. Among the 48 activators identified (Fig. 1D), we observed that 20 were implicated in WNT pathways, either directly (based on published evidence) or through BioGRID (*29*) interactions (extended data 1). This led us to hypothesize that WNT may play a role in regulating EV uptake.

Given that WNT ligands are known to decorate EVs and contribute to their functional roles (*14*, *22*), we next investigated whether the uptake of WNT-decorated EVs differs from that of WNT-naïve EVs. We also wanted to compare canonical (Wnt3A) and noncanonical (Wnt5A) ligands, given the potential differences in their molecular mode of action.

**Fig. 2.**
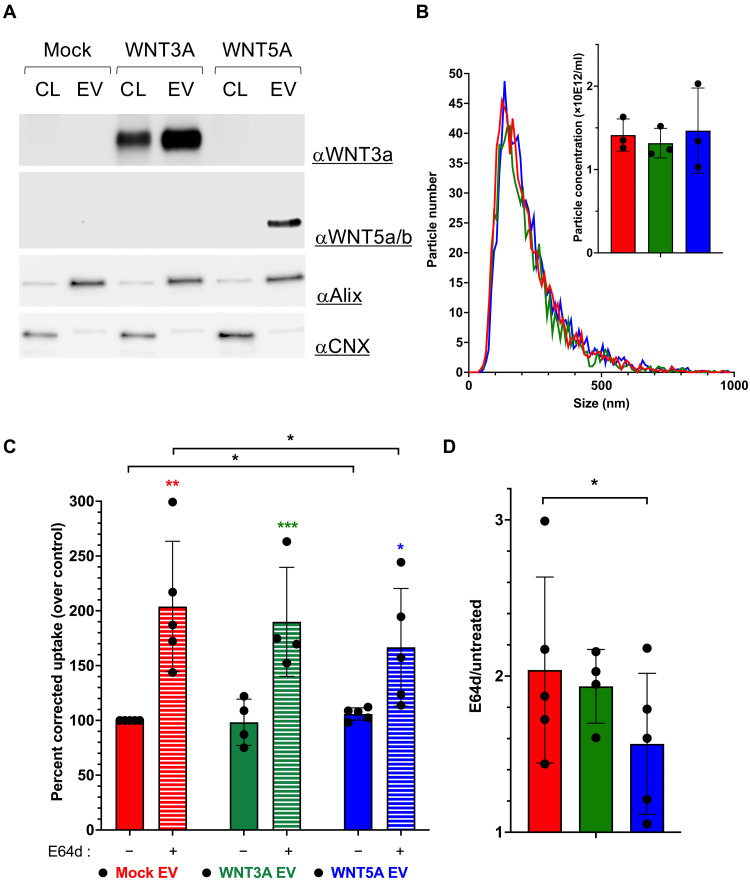
Wnt5A-EVs are diverted from lysosomal degradation. (**A**) HEK293 cells were cotransfected with NLCD63 along with plasmids encoding WNT3A, WNT5A, or a mock control. Equivalent amounts of proteins from cell lysates (CL) or EVs produced from these cells were subjected to Western blotting with antibodies directed against the indicated proteins. CNX, calnexin. (**B**) Nanoparticle tracking analysis was performed on each EV preparation, and results concerning particle size and concentration were plotted. (**C**) Corrected EV uptake values were obtained for EVs as in Fig. 1 in the absence or presence of 20 mM E64d (hatched bars) for 24 hours. (**D**) The E64d/untreated (UT) ratio represents the EV uptake values from (C) in the presence of E64d divided by their dimethyl sulfoxide control counterparts (UT). *P < 0.1; **P < 0.01; ***P < 0.001. When colored, * stipulate comparisons within the same EV type. When black and above brackets, * stipulate comparisons between different types of EVs.

To decorate EVs with specific WNTs, we overexpressed either Wnt3A or Wnt5A in human embryonic kidney (HEK) 293 cells, which are WNT naïve (*30*). As anticipated, we observed respective enrichment of Wnt3A or Wnt5A on EVs produced by these cells (Fig. 2A). Furthermore, we confirmed that WNT-decorated EVs did not differ significantly from WNT-naïve EVs in terms of size or production yield (Fig. 2B).

When applied to HeLa acceptor cells, we observed a slight increase in the uptake of Wnt5A-decorated EVs (which also contained the luminescent marker NLCD63 for detection purposes; fig. S2A). This was accompanied by a decreased accumulation of these EVs in the presence of lysosomal protease inhibition (E64d; Fig. 2C). Consequently, the E64d/untreated (UT) ratio was significantly lower for Wnt5A-EVs (Fig. 2D), suggesting that these EVs are less degraded by lysosomes compared to WNT-naïve (mock) or Wnt3A-EVs upon uptake.

Because HeLa cells, like other cancer cell lines, are known to express some WNT ligands, we tested whether decorating HeLa EVs with Wnt3A or Wnt5A would have the same effect as when decorating EVs from WNT-naïve HEK293 cells. We observed that, as for HEK293 cells, Wnt3A or Wnt5A were enriched in HeLa EVs when donor cells overexpressed them (fig. S2B). Furthermore, Wnt5A-decorated EVs from HeLa cells were also less degraded by lysosomes compared to mock-EVs or Wnt3A-EVs (fig. S2C).

Additionally, we tested whether the luminescent EV reporter used had an impact on our observations. We observed that, when we used a different, broader EV marker [namely, NLHsp70 (*10*, *11*)] as uptake reporter, we still observed that Wnt5A-bearing EVs were less directed to lysosomes than their WNT naïve counterparts (fig. S3).

In conclusion, using two different EV donor cells (HEK293 and HeLa) and two different EV markers (NLCD63 and NLHsp70), we demonstrated that the presence of Wnt5A on EVs dictated their escape from lysosomal degradation, suggesting that our observation is little restricted by biological context.

**Fig. 3.**
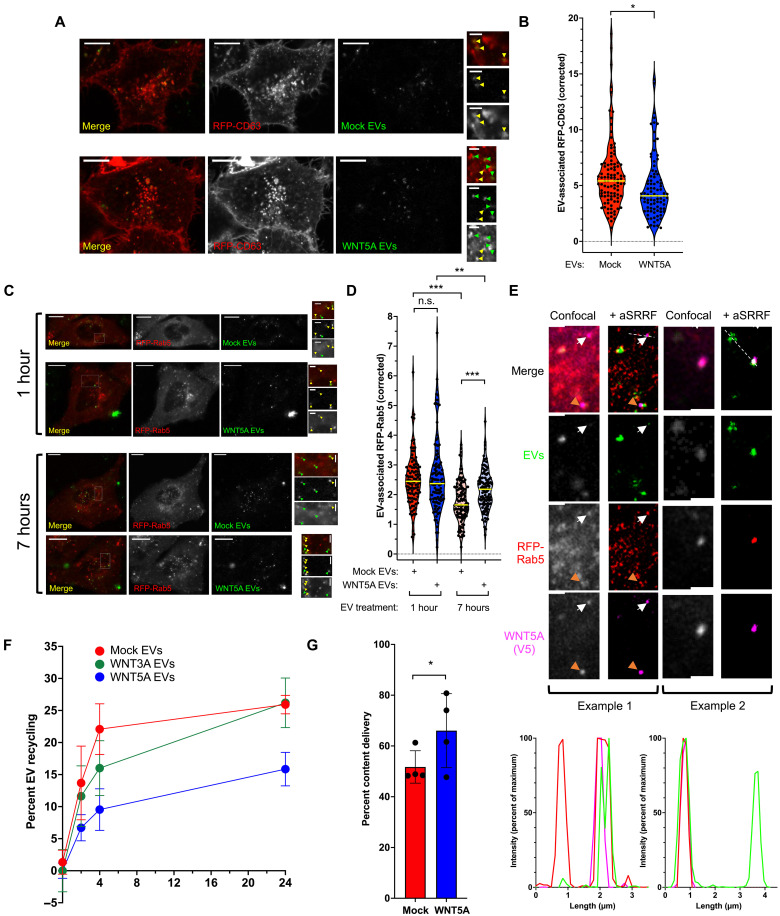
WNT5A-EVs follow a distinct uptake route. (**A**) GFP-EVs were produced from HeLa^GFPHSP70^ cells transfected with plasmids encoding WNT5A or a mock control (fig. S3). Recipient HeLa cells were transfected with RFP-CD63. The indicated EVs were incubated with the recipient cells for 7 hours before being imaged. Insets from the main images are shown on the right. (**B**) The corrected RFP-CD63 signal associated with GFP-EV puncta was quantified for at least 90 events. (**C**) EVs as in (A) were incubated with RFP-Rab5–transfected recipient HeLa cells for 1 or 7 hours before being imaged. Insets from the main images are shown on the right. (**D**) The corrected RFP-Rab5 signal associated with GFP-EV puncta was quantified for at least 100 events. Yellow arrowheads indicate colocalized puncta, and green arrowheads indicate EVs that do not colocalize with the red marker. (**E**) GFP-EVs were produced from HeLa^GFPHSP70^ cells transfected with a plasmid encoding WNT5A-V5 and incubated with RFP-Rab5–transfected recipient HeLa cells for 7 hours before being processed for anti-V5 immunofluorescence. Two example insets from imaged cells are shown before (confocal) and after aSRRF processing (full images in fig. S5). Line plots (merge) are shown below each example with intensity quantification of each fluorescent marker. In example 1, the white arrow shows a dot with full colocalization of all markers, and the orange arrowhead shows segregation of GFP-EVs from the others. (**F**) EVs as in Fig. 2 were tested for recycling for the indicated time. (**G**) EVs produced from HEK293 cells cotransfected with NLHSP70 (for luminescent detection) along with plasmids encoding WNT5A or a mock control were tested for content delivery. *P < 0.1; **P < 0.01; ***P < 0.001; n.s., not significant. Scale bars represent 10 mm for images and 2 mm for insets.

To determine whether Wnt5A-decorated EVs escaped lysosomal degradation through rerouting to other organelles in recipient cells, we decided to observe the fate of uptaken EVs through confocal microscopy. To do this, we first isolated fluorescently tagged EVs (*10*) from HeLa^GFPHSP70^ cells that were transfected with or without Wnt5A (fig. S4). We observed that, after 7 hours of uptake, the Wnt5A-decorated EVs (also containing GFP-Hsp70 for visualization) colocalized less with the late endosomal marker red fluorescent protein (RFP)–CD63 than WNT-naïve EVs in recipient cells (Fig. 3, A and B), confirming that they are diverted away from late endosomes/lysosomes. Although at an earlier uptake time point (1 hour) both WNT-naïve and Wnt5A-decorated EVs are associated with the early endosome marker RFP-Rab5 (Fig. 3, C and D), we observed that this association persist after 7 hours for Wnt5A-decorated EVs, whereas WNT-naïve EVs lose their RFP-Rab5 colocalization. To increase the resolution of our confocal images, we applied the augmented super-resolution radial fluctuation (aSRRF) technique (*31*) that allowed us to observe that uptaken WNT5A-decorated EVs could be found in 500- to 600-nm Rab5-positive structures (Fig. 3E). We conclude that Wnt5A-decorated EVs remain associated with early endosomes while WNT-naïve EVs progress through the endocytic pathway all the way to late endosomes/lysosomes, where they end up being degraded for the most part.

If Wnt5A-decorated EVs linger in early endosomes and do not proceed along to lysosomes, then what may their fate be? We first tested whether they could be recycled back to the extracellular medium. However, we observed that this was not the case, as Wnt5A-decorated EVs were less recycled than their naïve or Wnt3A-decorated counterparts (Fig. 3F).

An alternate possibility is that EV cargoes are released into the recipient cells. We show that Wnt5A-decorated EVs release more of their content into the cytoplasm of acceptor cells compared to their WNT-naïve counterparts (Fig. 3G). In conclusion, we demonstrate that Wnt5A-decorated EVs follow a distinct uptake route once internalized: They are diverted from lysosomes and linger in early endosomes from which they deliver more of their content into the cytoplasm of acceptor cells.

**Fig. 4.**
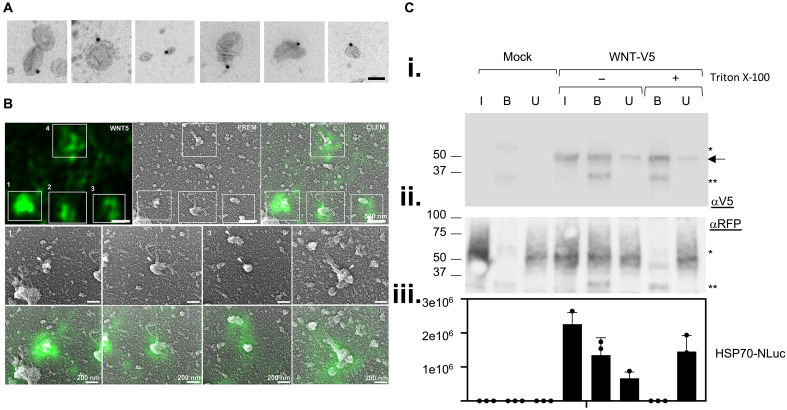
WNT5A-decorated EVs are redirected by the cognate receptor RYK. (**A**) Representative images of anti-V5 immunogold labeling of EVs isolated from HeLa cells expressing WNT5-V5. (**B**) Correlative light PREM (platinum replica electron microscopy) was used to localize WNT5-V5 at a nanoscale resolution. WNT5-V5 was identified using anti-V5 immunofluorescence (green) and aligned with the corresponding PREM replica of the same region. On the top, an overview of fluorescence, PREM, and merged CLEM (correlative light and electron microscopy) images is presented. Boxed regions highlight regions of interest shown at higher magnification in panels 1 to 4. On the bottom, higher-magnification PREM images (top) are displayed alongside the corresponding CLEM overlays (bottom). Arrowheads indicate membrane-associated vesicular structures that spatially coincide with WNT5-V5 fluorescence puncta. (**C**) EVs were isolated from HeLa cells expressing RFP-CD63 and NLuc-HSP70, two generic EV markers, and coexpressing either WNT5-V5 or not (mock). EVs were immunoisolated with an anti-V5 antibody and Protein G–coupled beads in the absence or presence of Triton X-100. (i and ii) Immunoblots showing the V5 and RFP signals in 40% input (I) (i), 90% bound fraction (B), and 80% unbound fraction (U). Panel (i) is representative of four independent experiments and panel (ii) of two independent experiments. * Antibody (Ab) heavy chain; ** Ab light chain; the arrow in (i) indicates specific signal. (iii) Luminescence signal from 1% of each fraction, reflecting the distribution of NLHSP70. Scale bar represents 100 nm for electron micrographs.

Our screening identified RYK as one of the most potent regulators of EV uptake (Fig. 1D and extended data 1). RYK is a well-established Wnt5A co-receptor (*32*). We therefore hypothesized that Wnt5A exposed on the surface of EVs would engage its cognate receptor RYK on the surface of acceptor cells, leading to a rerouting of the EVs. Considering that the mechanism of Wnt protein secretion on EVs and, therefore, their topology is poorly described and somewhat up for debate (*33*, *34*), we decided to first test whether Wnt5A was accessible for RYK interaction on the surface of EVs. To do this, we first performed anti-V5 immunogold labeling of EVs produced from active Wnt5A-V5 (*35*)–expressing HeLa cells. We could observe that the surface of both smaller (<50 nm in diameter) and larger (>200 nm diameter) vesicles were decorated with Wnt5A-V5 (Fig. 4A). In addition, correlative light and platinum replica electron microscopy allowed us to visualize the 3D topography of Wnt5A-V5–decorated EVs (Fig. 4B) and confirm that, although EVs of various sizes are Wnt5A decorated, not all structures are. Last, we observed that we could immunoisolate these EVs using an anti-V5 antibody in a specific manner (Fig. 4C) as we could detect both an EV membrane-associated marker (RFP-CD63) and an intraluminal EV marker (NLHsp70) in the immunoisolate. This coimmunoisolation, as expected, was lost in the presence of Triton X-100, which disrupts EV membranes. Together, these results suggest that Wnt5A is exposed on the surface of EVs in our experiments. As has been observed in Fig. 4 (A and B), these data indicate that Wnt5A-positive EVs represent only a fraction of the bulk isolated EV population as more than 50% of classical EV markers such as CD63 and HSP70 were not coimmunoisolated.

**Fig. 5.**
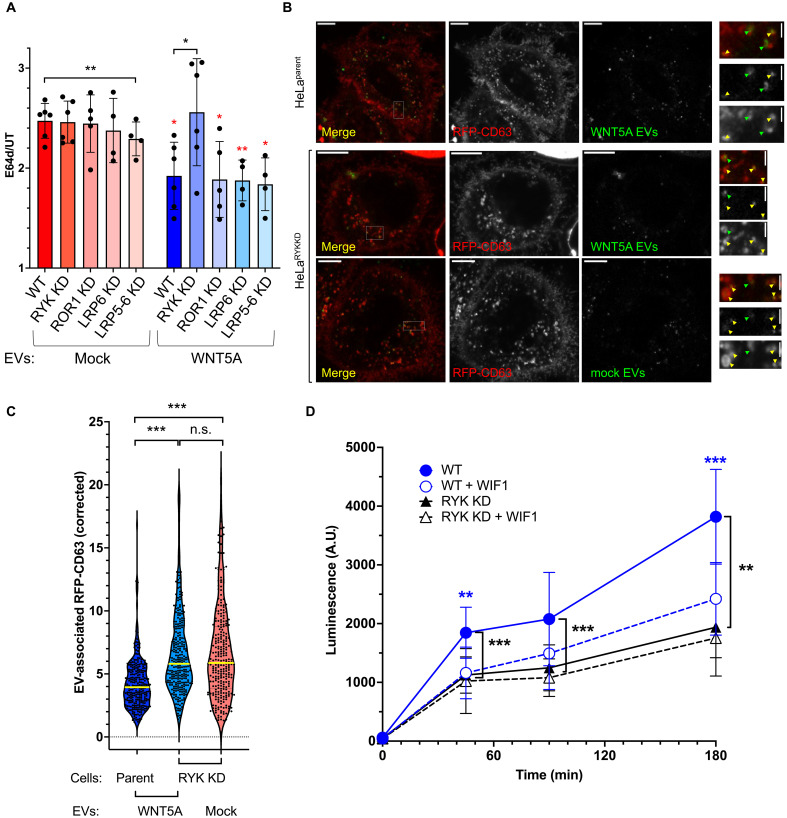
WNT5A-decorated EVs are redirected by the cognate receptor RYK. (**A**) EV uptake was performed as in Fig. 2C on parent HeLaCRISPR [wild type (WT)] or on recipient cells that were knocked down (KD) for the indicated genes. The E64d/UT ratio was obtained as in Fig. 2D. (**B**) GFP-EVs produced from HeLaGFPHSP70 cells transfected with plasmids encoding WNT5A, or a mock control were incubated with RFP-CD63-transfected parental or RYK knockdown (RYKKD) recipient cells for 7 hours before being imaged. Insets from the main images are shown on the right. (**C**) The corrected RFP-CD63 signal associated with GFP-EV puncta was quantified for at least 270 events. Yellow arrowheads indicate colocalized puncta, and green arrowheads indicate EVs that do not colocalize with the red marker. (**D**) EV binding was tested at 4°C. Conditioned medium from cells producing luminescent Wnt5A-decorated EVs along with or without the secreted protein WIF1 was incubated on either WT or RYK KD recipient cells. At the end of the indicated incubation times, the luminescence remaining associated with cells was assessed. *P < 0.1; **P < 0.01; ***P < 0.001; n.s., not significant. Scale bars represent 10 mm for immunofluorescence images and 2 mm for associated insets. When colored, * stipulate comparisons within the same recipient cells. When black and above brackets, * stipulate comparisons between different recipient cells. A.U., arbitrary unit.

We next investigated the fate of Wnt5A-decorated EVs in cells depleted of RYK or of other known Wnt co-receptors. We engineered recipient cells that were knocked down for either RYK, ROR1, or LRP6 alone or along with LRP5 (fig. S6) and assessed EV uptake with and without E64d to test for lysosomal routing. Figure 5A shows that the decreased E64d/UT ratio of Wnt5A-EVs observed in wild-type recipient cells was corrected in RYK-depleted cells, suggesting that Wnt5A-EVs require RYK to be diverted from lysosomes. Notably, none of the other Wnt co-receptor tested had an impact Wnt5A-EVs rerouting, including the Wnt5A co-receptor ROR1. This finding is consistent with the lack of effect observed for ROR1 depletion in the screening (extended data 1), further confirming the specificity of RYK in regulating the lysosomal degradation of Wnt5A-EVs. LRP5/6 co-receptors, which are known functional Wnt3A co-receptors but also interact genetically and physically with Wnt5A (*36*, *37*), were tested as a simple LRP6 knockdown or a double LRP5/6 knockdown in the light of LRP5 compensatory overexpression in single LRP6 knockdown (fig. S6C) and known functional redundancy of these genes (*38*). Although LRP5/6 knockdown cells seem to show a slight deficiency in lysosomal degradation of Wnt-naïve EVs, it was not altered for Wnt5A-EVs, suggesting no Wnt5A-specific function of these co-receptors in this context.

Additionally, we observed that the reduced association of Wnt5A-EVs with the late endosomal marker RFP-CD63 after 7 hours of uptake was restored in RYK-depleted recipient cells (Fig. 5, B and C). This further supports the notion that RYK dictates the rerouting of Wnt5A-EVs away from lysosomes.

To tease out at which uptake stage RYK reroutes Wnt5A-bearing EVs, we performed an EV binding assay (fig. S7). Conditioned medium from donor cells expressing WNT5A and NLHsp70 was incubated with recipient cells at 4°C, a temperature at which internalization (endocytosis) is blocked but not protein-protein interactions. We observe that the binding of WNT5A-decorated EVs (traced through NLHsp70 activity) increases over time, and it is dependent on RYK expression as it is less abundant in RYK-depleted cells (Fig. 5D). Moreover, the expression of the Ryk-specific secreted Wnt inhibitory factor WIF-1 (*39*) also decreases Wnt5A-EV binding in wild-type cells while it has no impact in RYK-depleted cells (Fig. 5D).

**Fig. 6.**
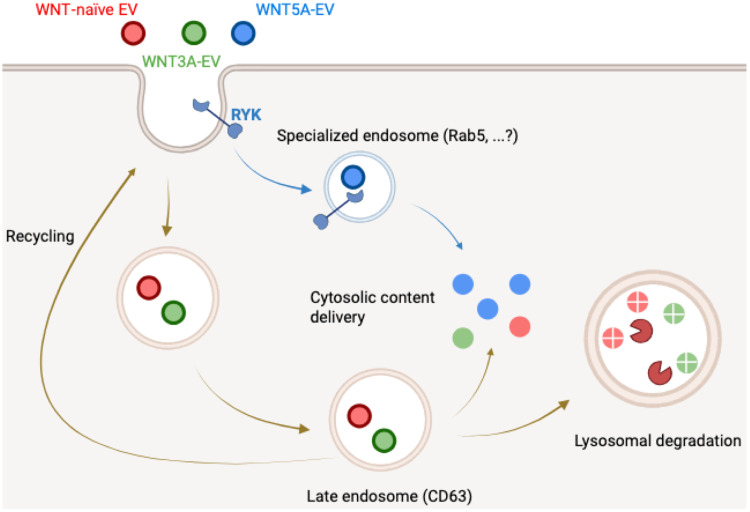
WNT5A-decorated EVs are specifically rerouted in acceptor cells. Once WNT-naïve or WNT3A-decorated EVs are internalized by acceptor cells, they follow the endocytic pathway all the way to lysosomes, which leads to more than half being degraded after 24 hours, some are being recycled back to the extracellular milieu and some deliver their content through endosomal escape. WNT5A-EVs are, however, diverted away into a different endosome population by specific interaction with RYK at the plasma membrane. These specialized endosomes are long-lived and show an increase in the cytosolic delivery of their content. Created in BioRender. Dancourt, J. (2026) https://BioRender.com/9c0k2e9.

These results strongly suggest that the rerouting of Wnt5A-decorated EVs occurs very early, at the level of specific ligand–co-receptor recognition at the plasma membrane of recipient cells and that these EVs might be internalized in specific endosomes that end up not fusing to lysosome (Fig. 6).

## DISCUSSION

By interrogating 300 genes identified as part of the dynamic surfaceome, coding for transmembrane proteins that cycle between the plasma membrane and endolysosomal compartments, we discovered that a significant proportion of EV uptake activators are associated with WNT pathways (Fig. 1).

While Wnt ligands have been shown to associate with EVs, little is known about the specific fate, if any, of Wnt-bearing EVs in recipient cells. We thus hypothesized that the involvement of WNT pathways in EV uptake revealed by our screening might be directly due to the presence of WNT ligands on EVs.

We observed that Wnt5A-EVs were specifically rerouted away from degradative compartments (Fig. 2). We also observed that Wnt5A-EVs were less recycled back to the extracellular space than WNT-naïve or even Wnt3A-EVs. Furthermore, the content of Wnt5A-EVs was delivered to the acceptor cell cytoplasm to a greater extent than that of WNT-naïve EVs (Fig. 3).

The subcellular localization of Wnt5A-EVs was assessed at various time points postuptake, revealing that they remained primarily in early Rab5-positive endosomal structures. In contrast, their WNT-naïve counterparts progressed from early to late endosomes (Fig. 3). This retention in early endosomes may account for the increased content delivery of Wnt5A-EVs as endosomal escape has been shown to be more efficient from early endocytic compartments (*40*). Our results and others’ (*11*, *12*) previously suggested that EV content delivery was favored by endosomal mild acidification. Such mild acidification occurs in early endosomes, which serve as the preferred site of delivery for model viruses such as vesicular stomatitis virus and SFV (semliki forste virus) (*41*), which require similar conditions to enable content delivery through membrane fusion. It remains unclear how “stagnant” early endosomes, those that do not mature into lysosomes, favor Wnt5A-EV content delivery. This could be explained simply by an increased number of potential fusion events due to extended residence time, or it may involve the elaboration of more complex signaling pathways through the establishment of signalosomes (*42*). This mechanism remains to be further elucidated.

The different trafficking of Wnt3A- and Wnt5A-EVs in recipient cells upon uptake begs the question of the role of signaling in these events as Wnt3A and Wnt5A theoretically engage different signaling events: canonical signaling for Wnt3A and noncanonical signaling for Wnt5A.

Activation of the canonical (β-catenin–dependent) WNT signaling pathway can be mimicked by the GSK3 inhibitor ChIR99021 (*43*, *44*). As expected, we observed that this drug induced Ser^675^ phosphorylation of β-catenin in our cells (fig. S8A) (*45*), which was associated with a slight increase in EV uptake (fig. S8B). This effect was only detectable after long EV uptake time points (24 hours), which, as discussed previously (fig. S1H), is affected by the natural lysosomal degradation of EVs. Notably, inhibition of lysosomal proteases by E64d markedly enhanced the effect of ChIR99021, as evidenced by the increased E64d/UT ratio (fig. S8C), indicating greater lysosomal delivery of EVs. Activation of the canonical WNT pathway has previously been shown to trigger macropinocytosis (*46*, *47*), and we confirm that this occurs in our cells as well (fig. S8D). Additionally, we observed that the increase in EV uptake upon this stimulation was inhibited by the macropinocytosis inhibitor EIPA (fig. S8E), suggesting that the canonical WNT pathway promotes EV uptake through the induction of macropinocytosis.

To test whether noncanonical WNT pathways affect EV uptake, at least through signaling regulation, we transfected recipient cells with Wnt5A, known to trigger non canonical WNT signaling (*48*), and performed an EV uptake assay. We observed an increase in the phosphorylation of the signaling effector Dvl2 in Wnt5A-transfected cells, indicating activation of noncanonical signaling (fig. S8F) (*49*, *50*). In the presence of lysosomal protease inhibition, we observed a decrease in the luminescence activity associated to the Wnt5A-transfected recipient cell (fig. S8G), suggesting reduced delivery to lysosomes, as evidenced by a lower E64d/UT ratio (fig. S8H).

Collectively, these results show that WNT pathways influence EV uptake through different mechanisms: The canonical pathway enhances EV macropinocytosis, whereas the noncanonical pathway appears to regulate the degradation of internalized EVs by lysosomes.

Notably, the small amount of EVs incubated with acceptor cells during EV uptake experiments (typically, 0.1 μg for 10,000 cells) was insufficient to trigger detectable signaling events at the cellular level (fig. S9). This lack of detectable signaling may explain why Wnt3A-decorated EVs do not induce their own macropinocytosis, unlike the effect observed upon β-catenin activation with ChIR99021 (compare Fig. 2D and fig. S8C). These findings suggest that the rerouting of Wnt5A-EVs may be independent of signaling events, relying instead on more direct interactions, such as with a specific co-receptor.

Wnt protein secretion and their association with EVs have been previously investigated and debated. How WNT is initially targeted to EVs remains to be fully elucidated, although recent studies suggest the existence of EV-targeting sequences and the involvement of coatomer complexes in a novel topological paradigm. Here, we focus exclusively on the downstream events and the impact of WNT on EV uptake, ensuring that WNT is exposed on the surface of EVs (Fig. 4). One of the most prominent activators identified during the screening was RYK, the cognate co-receptor for Wnt5A. We tested whether RYK played a crucial role in the rerouting of Wnt5A-EVs and demonstrated that it does. In RYK-depleted cells, the defect in lysosomal localization of Wnt5A-EVs was restored, both in terms of EV degradation and colocalization with the late endosomal marker CD63 (Fig. 5).

The fact that RYK stood out as an activator of EV uptake in the screening, which was performed with EVs derived from HeLa cells suggest that WNT5A was present on a portion of EVs containing our NLCD63 reporter, even at levels that were barely detectable by Western blots (fig. S2B). Although the proportion of Wnt5A-EVs in our EV population is difficult to assess, this suggests that the Wnt5A/RYK axis is highly sensitive and likely to be underestimated in the results that we are reporting.

We performed our assay using a cargo-based approach, tracking CD63 or HSP70 that are generic markers present in most EV subtypes, including Wnt5A-positive EVs, as suggested by our results. Further studies focusing on Wnt5A-EV content will be needed to determine whether the pathway that we describe provides a basis to formally identify a distinct EV subtype. At this stage, we can only speculate that Wnt5A-EVs follow a dedicated internalization route that may lead to specific cargo delivery or signaling events. Whether these EVs represent a bona fide subpopulation, defined as a distinct EV population harboring a characteristic ligand and specific cargoes associated with one or more biological functions, remains to be determined. The Wnt5A/RYK axis has been associated with several disease phenotypes, including cancer (*51*–*53*), and understanding whether EV rerouting participates in pathophysiology may open previously unexplored therapeutics avenues. For instance, the potency of engineered oncolytic EVs (*54*, *55*) could be increased if Wnt5-decorated EVs deliver more of their content to RYK-overexpressing cells, signature of several cancers.

Our results might also have an impact in terms of Wnt5A signaling modulation.

On the one hand, the increased macropinocytosis triggered by the WNT canonical signaling makes perfect sense in terms of the promotion of cell proliferation that this pathway is known for (*47*) as it first leads to signal amplification through GSK3 depletion (*56*) and then to increased lysosomal catabolism. We report here that EVs are also part of the extracellular material uptaken and degraded upon β-catenin signaling (fig. S8).

On the other hand, given that noncanonical signaling does not rely on the sequestering of an inhibitor as canonical signaling does (*56*), the derivation of EVs (that presumably bear the ligand) away from lysosomal degradation might serve as a signal amplification system, through signalosome stabilization (*57*). While a differential intracellular rerouting of a WNT co-receptor has previously been described (*58*), ours reports that Wnt5A signaling, contrary to β-catenin signaling, redirects ligand-bearing EV uptake to a less degradative route (fig. S8).

During the preparation of this manuscript, an article describing an independent genetic screening was published, identifying the endosomal COMMANDER complex as a key regulator of EV uptake (*59*). Notably, the components of the COMMANDER complex were not included in our screening library, as our design specifically excluded non–single-pass transmembrane proteins, preventing us from assessing their contribution in our system. We also note that this study did not identify any link to Wnt signaling pathways. This discrepancy may stem from methodological differences as their EVs were chemically modified at the surface for detection purposes, a modification that could potentially interfere with ligand-receptor interactions, including those involving Wnt5A and RYK.

Similarly, previous screening studies have reported a role for GPCRs in EV uptake, which were also absent from our library (*60*).

In conclusion, using an unbiased approach, we identified Wnt5A as a determinant of EV fate in acceptor cells. Wnt5A-bearing EVs are retained in early endosomes, whereas their Wnt3A- or Wnt-naïve counterparts continue along the endocytic pathway to lysosomal degradation. This specific rerouting occurs through the co-receptor RYK and leads to increased EV content release. Our study not only expands our molecular understanding of EV uptake but also introduces previously unexplored hypotheses regarding noncanonical WNT signaling mechanisms. While canonical Wnt signaling promotes general EV uptake by enhancing bulk macropinocytosis, RYK-dependent internalization provides a selective mechanism to prevent lysosomal clearance of Wnt5A-EVs. When combined, these mechanisms could offer an efficient way to control established WNT-dependent physiological functions through EV content delivery and potentiate signaling. Here, we use nonphysiologically relevant cell models to reveal and dissect the molecular mechanisms involved in EV uptake and delivery, and we identify the Wnt5A/RYK axis as a molecular determinant. However, it remains to be validated whether this mechanism represents a universal pathway for regulating EV uptake or whether it is specifically linked to well-established WNT functions. For instance, EVs have been proposed to serve as carriers of WNT, and other morphogens, to establish gradients during polarization and morphogenesis (*23*, *61*). Therefore, further investigation in more complex and integrated in vivo systems will be required to determine how the molecular mechanisms that we have characterized may contribute to the spatiotemporal regulation of core physiological functions.

For now, our results lead us to propose Wnt5A as a determinant of a loosely defined EV subtype that exhibits lysosomal rerouting. However, further studies aimed at characterizing their molecular content and putative functional machinery are required before these EVs can be considered bona fide subtypes, harboring specific molecular signatures connected to physiological functions.

## MATERIALS AND METHODS

### Cell culture

HeLa and HEK293 cells (American Type Culture Collection, Virginia, USA) and their transgenic derivatives were grown in high-glucose and pyruvate-containing Dulbecco’s modified Eagle’s medium (DMEM) (Gibco, Illinois, USA) complemented with 10% heat-inactivated fetal bovine serum (Biowest, France) at 37°C under 5% CO_2_ and high humidity.

A stable Cas9-expressing HeLa cell clone (HeLa^CRISPR^) was obtained by transfection of plasmid lentiCRISPRv2 neo (table S1) and clonal selection with geneticin (1 mg/ml; Gibco, Illinois, USA). The same strategy, using a plasmid encoding NLCD63, was used to obtain a stable NLCD63-expressing HeLa cell line (HeLa^NLCD63^). The stable GFPHSP70-expressing HeLa cell line (HeLa^GFPHSP70^) was described previously. Appropriate selection antibiotics were kept in culture medium for transgenic cell lines.

Transient transfections of plasmids (table S1) were performed using Lipofectamine 2000 (Invitrogen, Massachusetts, USA), and transient transfection of sgRNAs or small interfering RNAs (siRNAs) was performed using RNAiMAX Lipofectamine according to the manufacturer’s instructions. E64d and EIPA were from Sigma-Aldrich, and ChIR99021 was from Thermo Fisher Scientific.

### Arrayed CRISPR-Cas9 screening

#### sgRNA bank

A bank of chemically modified sgRNAs targeting the selected dynamic surfaceome genes was produced from Synthego (California, USA) who provided, for each gene, a pool of three custom-designed sgRNAs + EZ scaffold, as well as two negative controls (nontargeting sgRNAs) and two positive controls [RELA (rel-associated protein) positive and TRAC (T cell receptor alpha constant)] for screen quality assessment. The bank of sgRNA was stored and diluted as per the manufacturer’s instructions.

#### Screening workflow

HeLa^CRISPR^ cells were transfected with sgRNAs in triplicates on 96-well plates using 1 μl of 2 μM sgRNA and 0.3 μl of RNAiMAX Lipofectamine (optimal parameters obtained from fig. S1D). The bank was divided in six sets (50 genes per set). For each set, 2 days after transfection, the lipofection triplicate was split in two so that one triplicate was submitted to the EV uptake assay the next day and the other triplicate the day after. In Fig. 1B, one “plate” corresponds to one triplicate. In total, for each gene, a total of six corrected EV uptake values were obtained. Cells were assayed for EV uptake when reaching 80 to 90% confluency. For the EV uptake assay, EVs from HeLa^NLCD63^ cells were isolated and used fresh (stored less than 1 week at 4°C) as described below and applied on cells at 5 × 10^8^ to 10 × 10^8^ particles/ml for 4 hours. AlamarBlue HS reagent was added to each well at 10% (v/v) for the last 2 hours of the experiment. AlamarBlue HS and EV uptake values (see below) were then obtained for each well and expressed as percent of the average of all points of each plate (Fig. 1B). Corrected EV uptake values, expressed as the ratio of EV uptake over alamarBlue, were obtained for each gene and outliers were excluded (interquartile range method). Student’s *t* test and log_2_ fold change was obtained from all six values for each gene (outliers excluded).

### CRISPR efficiency assay

To assess the efficiency of genome editing on HeLa^CRISPR^, we used the TRAC sgRNA control (Synthego, California, USA) following the manufacturer’s instructions. Amplified genomic DNA was sequenced and ICE (intersection control evaluation) analysis (https://ice.editco.bio/#/) was performed to assess the knockout score. To test the efficiency of the screening workflow in fig. S1E, five sgRNA pools from the dynamic surfaceome bank were tested in the same way.

### EV isolation

EV donor cells were transfected with the indicated plasmids for 16 hours before being serum deprived for 20 hours. Conditioned medium was harvested and submitted to a 2000*g* centrifugation for 20 min at 4°C and then to a 100,000*g* ultracentrifugation for 1 hour 30 min at 4°C (45Ti rotor and Optima XE-90 ultracentrifuge, Beckman Coulter, California, USA). The resulting EV pellet was resuspended in 5 ml of DPBS (Dulbecco’s phosphate-buffered saline) and centrifuged 1 hour 30 min at 100,000*g* at 4°C (SW55 rotor and Optima XE-90 ultracentrifuge, Beckman Coulter, California, USA). The washed pellet was resuspended in <100 μl of DPBS, and EVs were either stored at −20°C (if destined to Western blot or particle metrics analysis) or immediately applied on acceptor cells (less than a week storage at 4°C).

### EV immunoisolation

HeLa cells were cultured in 24-well plates and transfected with plasmids encoding Cherry-CD63 and NLuc-HSP70, with or without WNT5-V5. Forty-eight hours posttransfection, conditioned media from three wells per condition were collected and subjected to sequential ultracentrifugation. The final 100,000*g* pellet was resuspended in 50 μl of phosphate-buffered saline (PBS), of which 25% was reserved for Western blot analysis and 1% for luminescence measurements to asses input content (I). Magnetic Protein G Dynabeads (50 μl; Thermo Fisher Scientific) were washed in ice-cold PBS and incubated with anti-V5 antibody (1 μl/ml) for 2 hours at 4°C. Beads were washed, and EVs containing or lacking WNT5-V5, were incubated with the antibody-coupled beads for 2 hours at 4°C in the absence or presence of 0.5% Triton X-100 to respectively test the specificity of V5-positive EV capture and distinguish direct EV pulldown (i.e., coimmunoisolation) from putative indirect V5-protein interactions (coimmunoprecipitation). The unbound fraction (U) was collected (80% for Western blot, 1% for luminescence), and beads were washed twice with PBS. Bead-bound fractions (B) were analyzed by Western blot (95%) and luminescence (1%).

### EV uptake assay

EVs were obtained from donor cells that were either HeLa^NLCD63^ stable cells (for the screening) or HeLa^CRISPR^ or HEK293 cells transiently cotransfected with NLCD63 and mock, Wnt3A, or Wnt5A plasmids (table S1). Acceptor HeLa cells were seeded in 96-well plates to reach 80 to 90% confluency on the day of EV incubation. The same amount of EV-associated luminescence for each condition was incubated with acceptor cells for the indicated time. After incubation, acceptor cells were washed twice in DPBS, and NL activity was measured in each well using the Nano-Glo Luciferase Assay System (Promega, Wisconsin, USA) following the manufacturer’s instructions using the iD3 SpectraMax microplate reader (Molecular Devices, California, USA). Unless otherwise noted, corrected EV uptake values (shown as percent of control) were obtained by correcting each uptake value by the alamarBlue value that corresponds to the same cells as for the genetic screening.

### EV binding assay

Donor HeLa cells were cotransfected with NLHsp70- and Wnt5A-expressing plasmids with or without a WIF-1 expression vector. After transfection, culture medium was replaced with serum-free DMEM for 20 hours. This conditioned medium was then subjected to centrifugation at 2000*g* for 20 min to remove cell debris. Luminescence activity was measured and normalized before this cleared conditioned medium was incubated with acceptor cells (either HeLa wild-type or RYK KD) on ice for the indicated time. After incubation, acceptor cells were washed twice with DPBS before being assessed for luminescent activity using the Nano-Glo Luciferase Assay System (Promega, Wisconsin, USA).

### EV recycling assay

EVs were obtained from donor HEK293 cells transiently cotransfected with NLCD63 and mock, Wnt3A, or Wnt5A plasmids and incubated on HeLa^CRISPR^ acceptor cells for 24 hours. Cells were then washed in DPBS and incubated in fresh medium (without phenol red as it interfered with luminescence readings). NL activity was measured in medium as well as in cells at the indicated time points using the Nano-Glo Luciferase Assay System (Promega, Wisconsin, USA) following the manufacturer’s instructions using the iD3 SpectraMax microplate reader (Molecular Devices, California, USA). Percent of recycling was calculated as the ratio of medium-associated luminescence divided by the total luminescence (medium + cells). Values at *t*_0_ were deduced.

### EV content delivery assay

EVs were obtained from donor HEK293T cells transiently cotransfected with NLHSP70 and either mock or Wnt5A plasmids (table S1). The same amount of EV-associated luminescence for each condition was incubated with HeLa^CRISPR^ acceptor cells for 24 hours before EV content delivery was performed as published (*10*). Briefly, cells were washed once and detached in DPBS before being submitted to lysis by multiple 20-gauge needle cycles and sonication. Unbroken cells were pelleted at 400*g* for 10 min at 4°C, and supernatant was spun at 100,000*g* for 1 hour at 4°C. The 100,000*g* supernatant was kept as the cytosolic fraction and the pellet as the membrane fraction. The percentage of content delivery was calculated as the ratio of luminescence found in the cytosolic fraction divided by the total luminescence (cytosol- + membrane-associated). As a control for each experiment, a sample of acceptor cells was transiently transfected with the membrane-bound NLCD63 and processed the same way. As previously published, this control revealed that membrane contamination in the cytosolic fraction did not exceed 20% (18.8 ± 4.2%).

### AlamarBlue HS cell viability assay

Cells were incubated for 2 hours in alamarBlue HS (Invitrogen, Massachusetts, USA) and its fluorescent signal measured according to the manufacturer’s instructions using the iD3 SpectraMax microplate reader (Molecular Devices, California, USA).

### Nanoparticle tracking analysis

Nanoparticle tracking analysis was performed using the ZetaView QUATT (Particle Metrix, Meerbusch, Germany) and its corresponding software (ZetaView 8.02.28) as described previously (*55*). Briefly, 1 ml of sample, diluted in DPBS, was loaded into the cell, and the instrument measured each sample at 11 different positions. After automated analysis of all positions and removal of any outlier positions, the size distribution of the particles was obtained.

### Western blot

Cells to be analyzed were scraped on ice in DPBS and pelleted at 1000*g* for 5 min at 4°C. Cell pellets were resuspended in PBS (phosphate buffered saline) lysis buffer [DPBS, Triton X-100 1%, EDTA-free protease/phosphatase inhibitor cocktail (Roche, Switzerland)] and incubated on ice for 10 min with intermittent vortexing. Samples were then submitted to a 15,000*g* centrifugation for 10 min at 4°C to pellet nuclei and unbroken cells. Supernatants (cell lysates) were collected. Protein concentration of cell lysate and EVs were obtained using the Micro BCA Protein Assay kit (Thermo Fisher Scientific, Illinois, USA). Unless otherwise noted, the same amount of protein was loaded in each lane of a gel to compare expression levels of the proteins of interest or to study protein enrichment in the case of comparison of cell lysates and EVs. Samples were mixed with Laemmli buffer (Bio-Rad, France) containing 10% β-mercaptoethanol, except for CD63 and CD9 detection (no β-mercaptoethanol) and loaded on 4 to 15% polyacrylamide gels (Bio-Rad, France). After electrophoresis, proteins were transferred onto polyvinylidene difluoride membranes using the Trans-Blot Turbo system (Bio-Rad, France). Membranes were incubated with DPBS containing 0.05% Tween 20 and 5% nonfat milk (blocking buffer) for 30 min at room temperature and then with a 1/1000 dilution of primary antibody [α-actin (catalog no. MAB1501, Millipore, Germany), α-calnexin (catalog no. ab133615, Abcam, UK), α-calreticulin (catalog no. ab22683, Abcam, UK), α-CD63 (catalog no. 556019, BD Bioscience, New Jersey, USA), α-Flag (F3165, Sigma-Aldrich, Missouri, USA), α-ALIX (clone3A9, 2171, Cell Signaling Technology, Massachusetts, USA), α-HEG1 (catalog no. ab121343, Abcam, UK), α-WNT5a/b (2530, Cell Signaling Technology, Massachusetts, USA), α-WNT3 (26744-1-AP, Proteintech, Illinois, USA), α-V5 (13202, Cell Signaling Technology, Massachusetts, USA), α-WIF1 (5652, Cell Signaling Technology, Massachusetts, USA), α-CD9 (CBL162, Sigma-Aldrich, Missouri, USA), α-Hsp70 (ADI-SPA-810-D, Enzo LifeScience, New York, USA), α-phospho-β-catenin (Ser^675^) (4176, Cell Signaling Technology, Massachusetts, USA), α-Dvl2 (3216, Cell Signaling Technology, Massachusetts, USA), α-LRP6 (3395, Cell Signaling Technology, Massachusetts, USA), and α-LRP5 (5731, Cell Signaling Technology, Massachusetts, USA)] in blocking buffer overnight at 4°C. Membranes were then washed and lastly incubated with a 1/5000 dilution of horseradish peroxidase (HRP)–coupled secondary antibody (α-mouse or α-rabbit, catalog no. 115-035-003, Jackson ImmunoResearch, UK) in DPBS containing 0.05% Tween 20 for 1 hour at room temperature. The HRP signal on membranes was developed using the Clarity Western ECL substrate (Bio-Rad, France) and imaged using the ImageQuant LAS 4000 (GE Healthcare Life Sciences, France).

### siRNA- and CRISPR-Cas9–mediated knockdown

siRNA directed against HEG1 and nontargeting siRNA (siGenome) were from Horizon Discovery (UK) and were used at 20 nM. HeLa cells were transfected with siRNAs using the RNAiMAX Lipofectamine reagent according to the manufacturer’s instructions and assessed for phenotype 3 days later.

For CRISPR-Cas9–mediated knockdown of RYK or ROR1, HeLa^CRISPR^ cells were transfected with plasmid pGL3-U6-sgRNA-PGK-puromycin (Addgene, Massachusetts, USA) into which a specific sgRNA sequence was cloned (fig. S6). Clones were obtained by puromycin selection and selected on the basis of gene expression level as assessed by quantitative polymerase chain reaction using the iTaq Universal One Step kit (Bio-Rad) with the following primers: forward (Fwd) 5′p-CCGCATACCTGAAAGATGGT-3′OH and reverse (Rev) 5′p-GAT CTA AGG CCC AGC AAC AG-3′OH for RYK; Fwd 5′p-TAATCGGAGAGCAACTTCA-3′OH and Rev 5′p-TGTAGTAATCAGCGGAGTAA-3′OH for ROR1. The PGK gene was used as loading control with primers Fwd 5′p-AGCTGCTGGGTCTGTCATCCT-3′OH and Rev 5′p-TGGCTCGGCTTTAACCTTGT-3′OH. Results were quantified using the ddCt (double delta Ct) method.

### Fluorescence imaging and image analysis

For EV colocalization studies, cells were imaged live under an LSM880 confocal microscope for no more than 15 min per sample. Image analysis was performed using the ImageJ software. Briefly, green channel puncta were detected using the “find maxima” function, and red channel fluorescence was measured for each punctum. The red channel fluorescence was then corrected by the average value of red signal in each cell analyzed (background corrected).

For dextran localization analysis, cells were loaded with lysine-fixable Texas Red 70-kDa dextran according to the manufacturer’s instruction (Invitrogen, Massachusetts, USA), washed in DPBS, and fixed for 15 min with 4% PFA (paraformaldehyde). Cells were then washed again and permeabilized with 0.02% (w/v) Triton X-100 for 10 min before being labeled with 4′,6-diamidino-2-phenylindole. Cells were then mounted in ProLong Diamond Antifade Mountant (Thermo Fisher Scientific, Massachusetts, USA) and left to cure overnight before imaging under an LSM880 confocal microscope.

For immunofluorescence, cells were washed in DPBS and fixed for 15 min with 4% PFA. Cells were then washed again and permeabilized with 0.02% (w/v) Triton X-100 for 10 min before being labeled with an 1/500 dilution of an anti-V5 antibody (13202, Cell Signaling Technology, Massachusetts, USA) in blocking buffer (1% bovine serum albumin) overnight at 4°C. Cells were then washed in DPBS and incubated with a 1/1000 dilution of a fluorescent secondary antibody (A-21071, Invitrogen, Massachusetts, USA) for 1 hour at room temperature before being washed again and mounted in ProLong Diamond Antifade Mountant (Thermo Fisher Scientific, Massachusetts, USA) and then left to cure overnight before imaging under an LSM880 confocal microscope.

aSRRF reconstruction (*31*) was applied to enhance the spatial resolution of fluorescently labeled EVs. Single confocal image was processed using aSRRF to generate synthetic image sequences, simulating temporal fluorescence fluctuations at the single-pixel level. For each pixel, a Gaussian distribution was defined, and 200 values were randomly sampled from this distribution to create an augmented image stack, which was subsequently subjected to SRRF analysis using the Fiji/ImageJ plugin with radiality magnification and temporal radiality averaging.

### Immunogold labeling of whole-mount EVs

Experiments (two sets) were performed as previously described (*62*). Briefly, EVs isolated from HeLa cells overexpressing Wnt5A-V5 were fixed in 2% PFA on ice before being adsorbed on electron microscopy grids. Grids were then washed, blocked, and immunolabeled with a rabbit anti-V5 antibody (13202, Cell Signaling Technology, Massachusetts, USA) before incubation with an anti-rabbit 15-nm gold conjugated antibody. Grids were then examined with a Jeol 1400 electron microscope operated at 120 kV and imaged with an Emsis Xarosa digital camera using Radius software (Münster, Germany).

### Correlative light and platinum replica electron microscopy

EVs isolated from HeLa cells overexpressing Wnt5A-V5 were placed on poly-l-lysine–coated, alpha-numerically gridded ibidi FluoroDishes, allowed to adhere for 15 min, and then fixed for 20 min in 4% PFA prepared in KHMgE buffer [70 mM KCl, 30 mM Hepes, 5 mM MgCl_2_, and 3 mM EGTA (pH 7.2)]. After rinsing, the samples were blocked for 30 min at room temperature in 1% bovine serum albumin in KHMgE buffer and then incubated with an anti-V5 primary antibody (13202, Cell Signaling Technology, Massachusetts, USA) for 2 hours at room temperature. Following washing, the samples were incubated with Alexa Fluor 488–conjugated secondary antibodies diluted 1:500 in KHMgE buffer for 1 hour. Fluorescence images were captured using spinning disk microscopy (Nikon) to identify regions of interest based on grid coordinates. After fluorescence imaging, the samples were further processed for platinum replica electron microscopy. Briefly, the samples were sequentially treated with 0.5% osmium tetroxide, 1% tannic acid, and 1% uranyl acetate, followed by graded ethanol dehydration and hexamethyldisilazane substitution. Dried samples were rotary shadowed with ∼2-nm platinum and 6-nm carbon. Platinum replicas were released from the glass coverslips using 5% hydrofluoric acid, extensively washed with distilled water, and collected onto 200-mesh formvar/carbon-coated electron microscopy (EM) grids. Electron microscopy imaging was conducted using a transmission electron microscope operated at 80 to 120 kV. Fluorescence and electron microscopy images were aligned using Adobe Photoshop. Grid coordinates facilitated the relocation of identical regions across imaging modalities, and alignment was further refined using fiduciary morphological landmarks to create correlative overlays of WNT5-V5 fluorescence signals onto individual vesicular structures. Images were adjusted for brightness and contrast and presented in inverted contrast.

### TOPFlash assay

HeLa cells were cotransfected with NLCD63 as a transfection control and either TOPFlash or FOPFlash. After treatment (with EVs and/or drugs), cells were washed and both NL and Firefly Luciferase activities were measured using the Nano-Glo Dual-Luciferase Reporter Assay System (Promega, Wisconsin, USA). Firefly Luciferase values were corrected by NL values to account for transfection efficiency differences and corrected FOP values were deduced from corrected TOP values to account for background activity of the reporter [results were thus plotted as (TOP/NL) − (FOP/NL)].

### Statistical analysis

Unpaired, two-tailed *t* test was performed using the Prism software (GraphPad, San Diego, California USA) on biological replicates. *P < 0.1; **P < 0.01; ***P < 0.001; ****P < 0.0001.

## Supplementary Materials

### The PDF file includes:

Figs. S1 to S9 Table S1 Legend for extended data S1

### Other Supplementary Material for this manuscript includes the following:

Extended data S1
