# Supplementary material for: Genetic screening identifies WNT5A/RYK as a determinant of extracellular vesicle fate
Source: Sci Adv. 2026 Jul 31;12(31):eaeb2877. doi: 10.1126/sciadv.aeb2877 (PMC13426412; doi:10.1126/sciadv.aeb2877)
Supplement: Supplementary file 1 — Figs. S1 to S9 Table S1 Legend for extended data S1 [file sciadv.aeb2877_sm.pdf]

Science Advances
AAAS

Supplementary Materials for

Genetic screening identifies WNT5A/RYK as a determinant of extracellular vesicle fate

Julia Dancourt *et al.*

Corresponding author: Julia Dancourt, julia.dancourt@ext.inserm.fr; Grégory Lavieu, gregory.lavieu@inserm.fr

Sci. Adv. **12**, eaeb2877 (2026)
DOI: 10.1126/sciadv.aeb2877

The PDF file includes:

Figs. S1 to S9
Table S1
Legend for extended data S1

Other Supplementary Material for this manuscript includes the following:

Extended Data S1

Figure S1. CRISPR/Cas9 screening optimization and verification

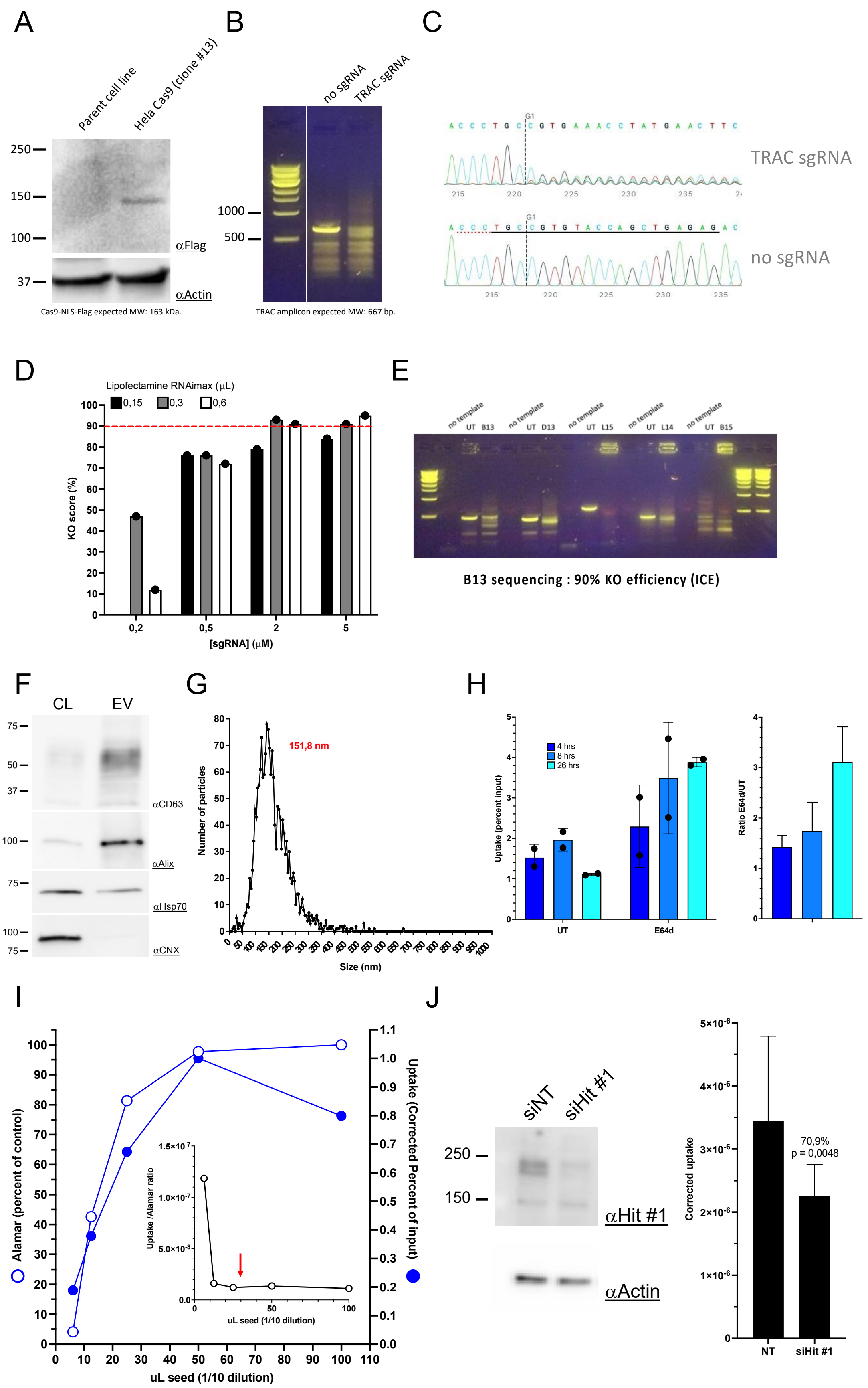

Figure S1 – Set-up of EV uptake CRISPR/Cas9 screening

A. Western blot analysis of parental HeLa as well as HeLa^{CRISPR} (HeLa Cas9 clone #13) showing that the latter stably expresses Cas9-NLS-Flag. Actin was used as a loading control. B. The TRAC sgRNA control (Synthego) was transfected into HeLa^{CRISPR} cells. 3 days later, genomic DNA was extracted and a region of the TRAC gene flanking the sgRNA target site was amplified by PCR. The amplicons were run on an agarose gel, which revealed a size change of the amplicon from the sgRNA-transfected cells, indicating efficient editing, which was confirmed by sequencing and ICE analysis (C). D. Dose-response experiment using different TRAC sgRNA and RNAiMAX Lipofectamine concentrations to assess KO score (ICE analysis) on HeLa^{CRISPR} in the experimental conditions of the arrayed screening. The dotted red line depicts our acceptability cutoff as an efficient KO score. E. 5 sgRNAs from the “dynamic surfaceome” bank (coordinates B13, D13, L15, L14, B15) were assessed as in B. All showed an amplicon size change, which was confirmed in one case to correspond to 90% KO efficiency. F. Western blot analysis of the same amount of protein from the cell lysate (CL) and EVs isolated from HeLa^{NLCD63} cells. G. Size distribution of the EVs in F obtained by Nanoparticle Tracking Analysis (NTA). The average size obtained was 151,8 nm. H. HeLa^{CRISPR} cells were incubated with EVs from F for the indicated times in the presence or absence of 20 μM E64d. EV uptake was measured (left) and an increase in the ratio of EV uptake in E64d-treated cells over control cells (E64d/UT, right) indicated that most EVs reached lysosomes at longer timepoints. I. HeLa^{CRISPR} cells were seeded at different dilutions and EV uptake was measured in parallel with Alamar blue in the same conditions as for the screening. The smaller plot shows that the EV uptake assay was linear over a wide range of cellular confluency (only the lowest being out of linear range). The red arrows point to the seeding used for the full-scale screening. J. SiRNA-mediated knockdown of HEG1 (Hit #1), validated at the protein level by western blot (left panel), resulted in almost 30% decreased EV uptake (right panel). P value was obtained by a two-tailed unpaired student test on 3 independent triplicates.

Figure S2. EVs produced from HeLa phenocopy EVs produced from HEK293

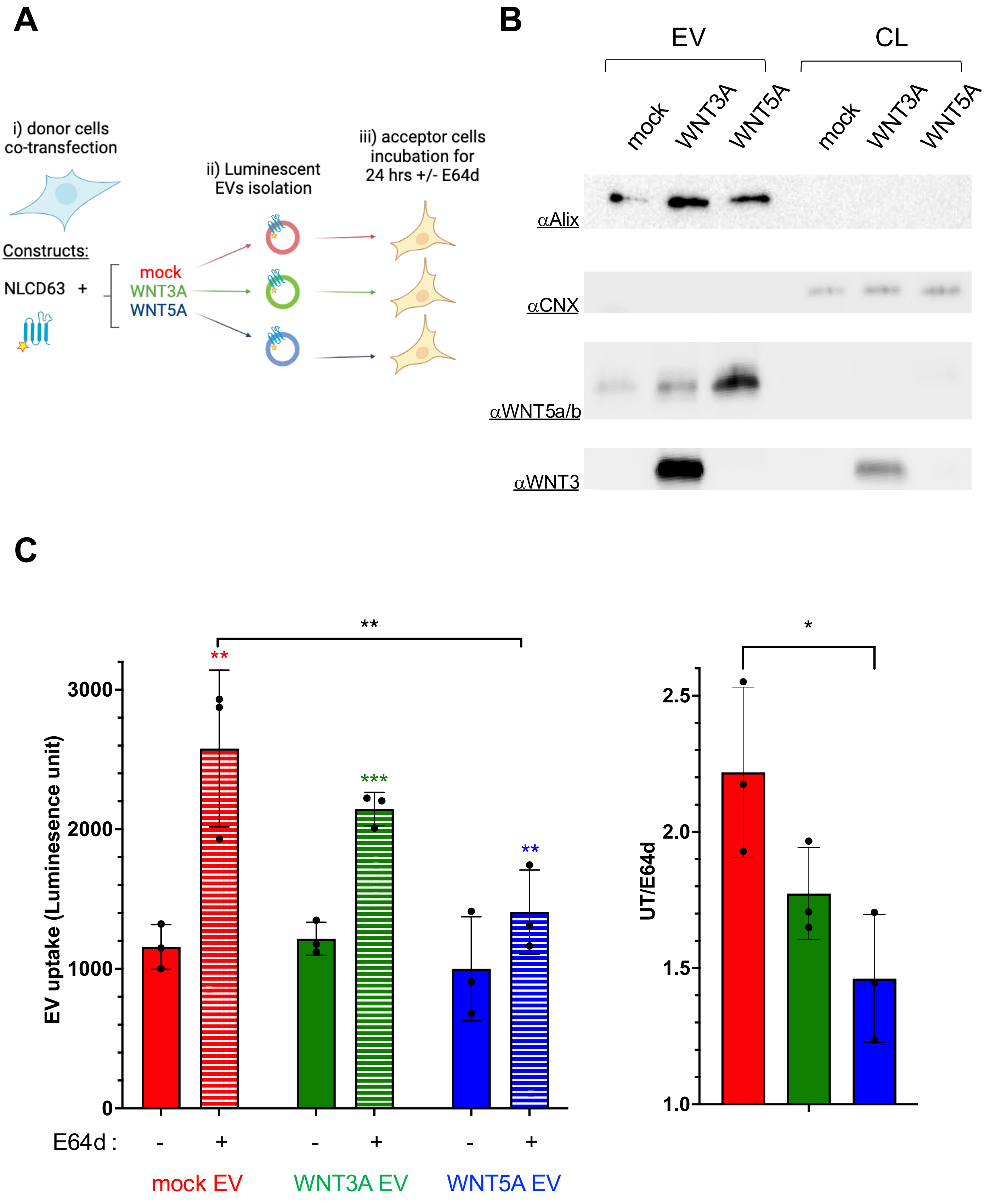

Figure S2 - EVs produced from HeLa phenocopy EVs produced from HEK293

A. Scheme for EV uptake experiments: EV donor cells (HEK293 or HeLa where indicated) were co-transfected with NLCD63 (for luminescent detection) along with plasmids encoding WNT3A, or WNT5A or a mock control (i), EVs were isolated (ii) and incubated with HeLa acceptor cells in the absence or presence of 20 μM E64d for 24 hours (iii). Created in BioRender. Dancourt, J. (2026) xnngj9u B. Equivalent amounts of proteins from HeLa donor cell lysates (CL) co-transfected with NLCD63 and the indicated plasmids, or EVs produced from these cells were subjected to western blotting with antibodies directed against the indicated proteins. CNX: calnexin. C. EVs as in B were isolated and EV uptake values were obtained for the indicated EVs in the absence or presence of 20 μM E64d (hatched bars) for 24 hours (left panel). The E64d/UT ratio (right panel) was obtained from the same data. *: p<0,1, **: p<0,01, ***: p<0,001. When colored, * stipulate comparisons within the same EV type. When black and above brackets, * stipulate comparisons between different types of EVs.

Figure S3. The Wnt5A-dependent re-routing of EVs is observed with another marker

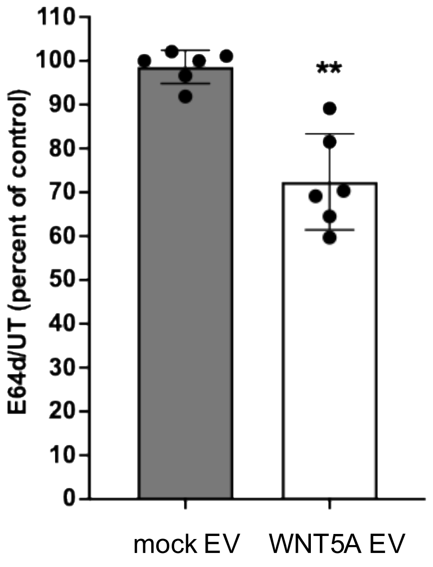

Figure S3 - The Wnt5A-dependent re-routing of EVs is observed with another marker
EVs were isolated as in Figure S2A except that NLHsp70 was used as an EV marker instead of NLCD63. EVs were then incubated with HeLa acceptor cells in the absence or presence of 20 μM E64d for 24 hours. EV uptake values were obtained for the indicated EVs and the E64d/UT ratio was plotted. **: p<0,01.

Figure S4. Characterization of EVs produced from HeLa^{GFPHSP70}

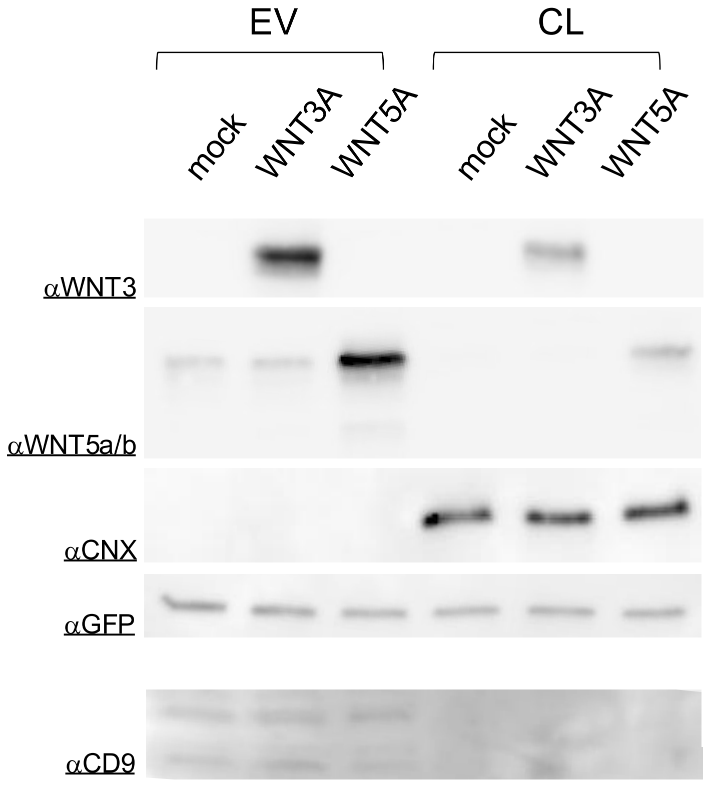

Figure S4 – Characterization of EVs produced from HeLa^{GFPHSP70}
HeLa^{GFPHSP70} were transfected with plasmids encoding WNT3A, WNT5A or a mock control. Equivalent amounts of proteins from cell lysates (CL) or EVs produced from these cells were subjected to western blotting with antibodies directed against the indicated proteins. CNX: calnexin.

Figure S5. High resolution co-localization of WNT5-V5-decorated EVs with RFP-Rab5 in recipient cells

Example 1

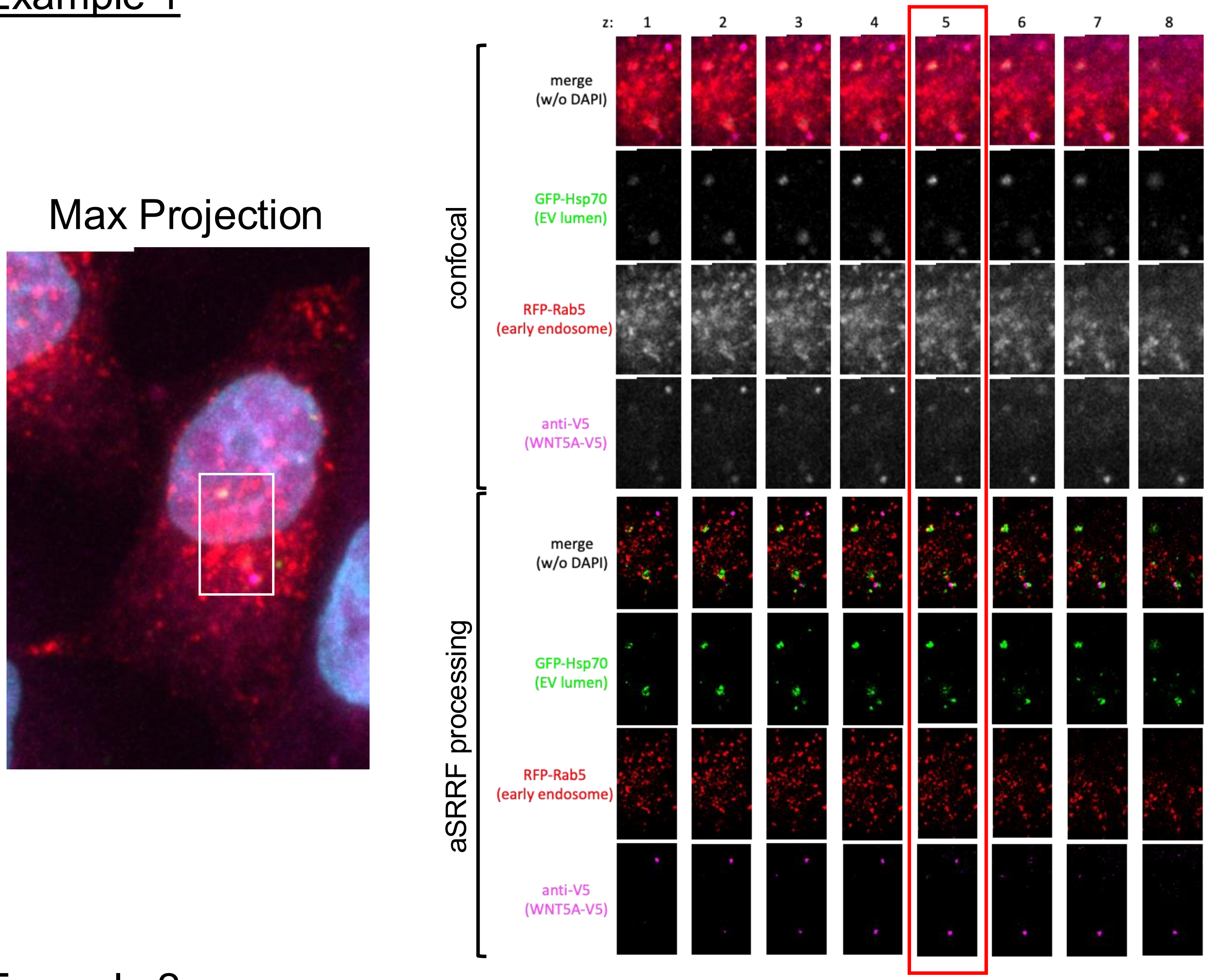

Example 2

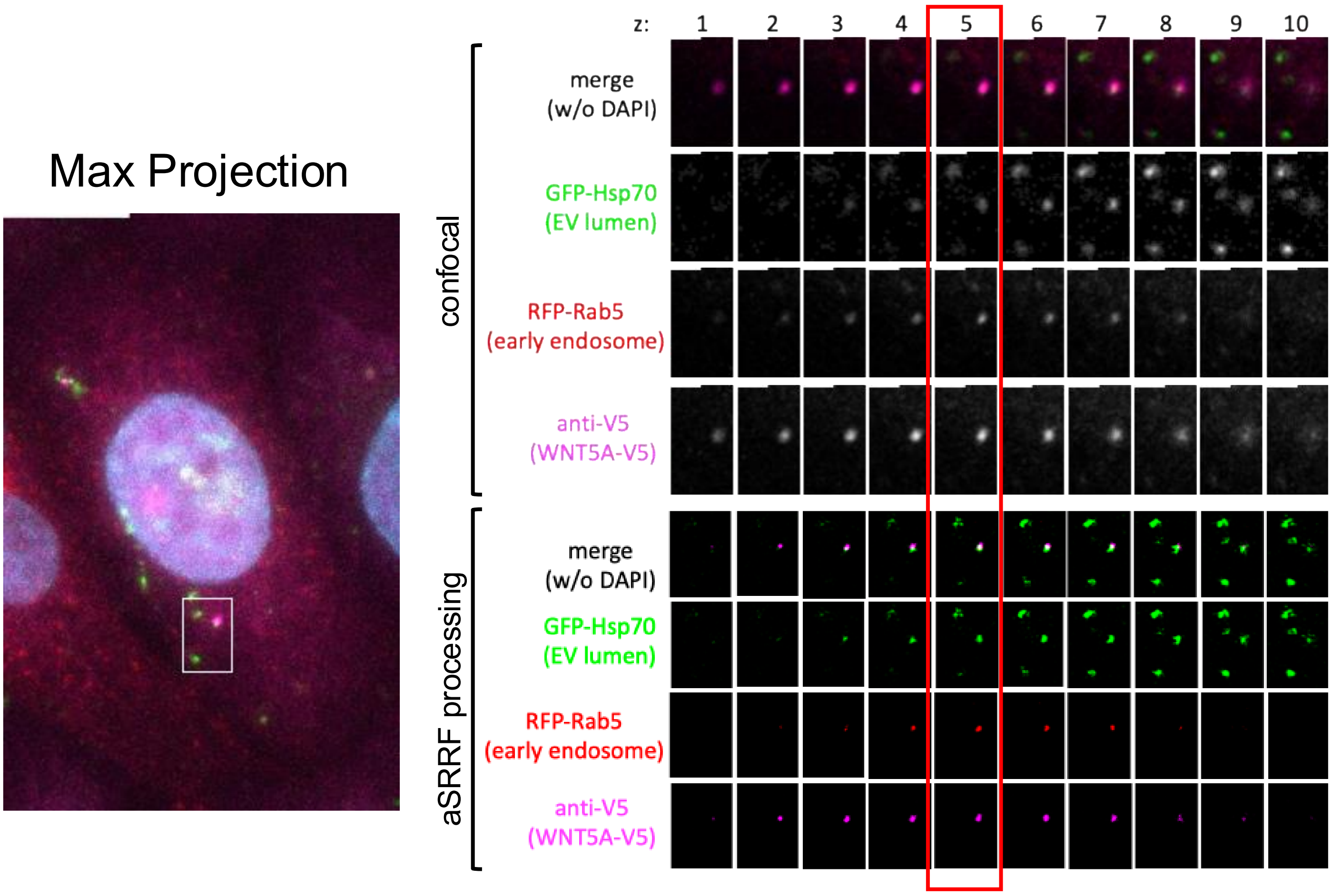

Figure S5 – High resolution co-localization of WNT5-V5-decorated EVs with RFP-Rab5 in recipient cells (related to Figure 2I)

GFP-EVs decorated with Wnt5A-V5 were incubated with RFP-Rab5-transfected recipient HeLa cells for 7 hours prior to being processed for anti-V5 immunofluorescence. Z-stack images acquired by confocal microscopy were post-processed by aSRRF to increase resolution. Images from z-slice number 5 are shown in Figure 2I. Maximum projection images show where the insets are. Scale bars are 10 μm for max projections and 2 μm for insets.

Figure S6. Knockdown cell lines characterization

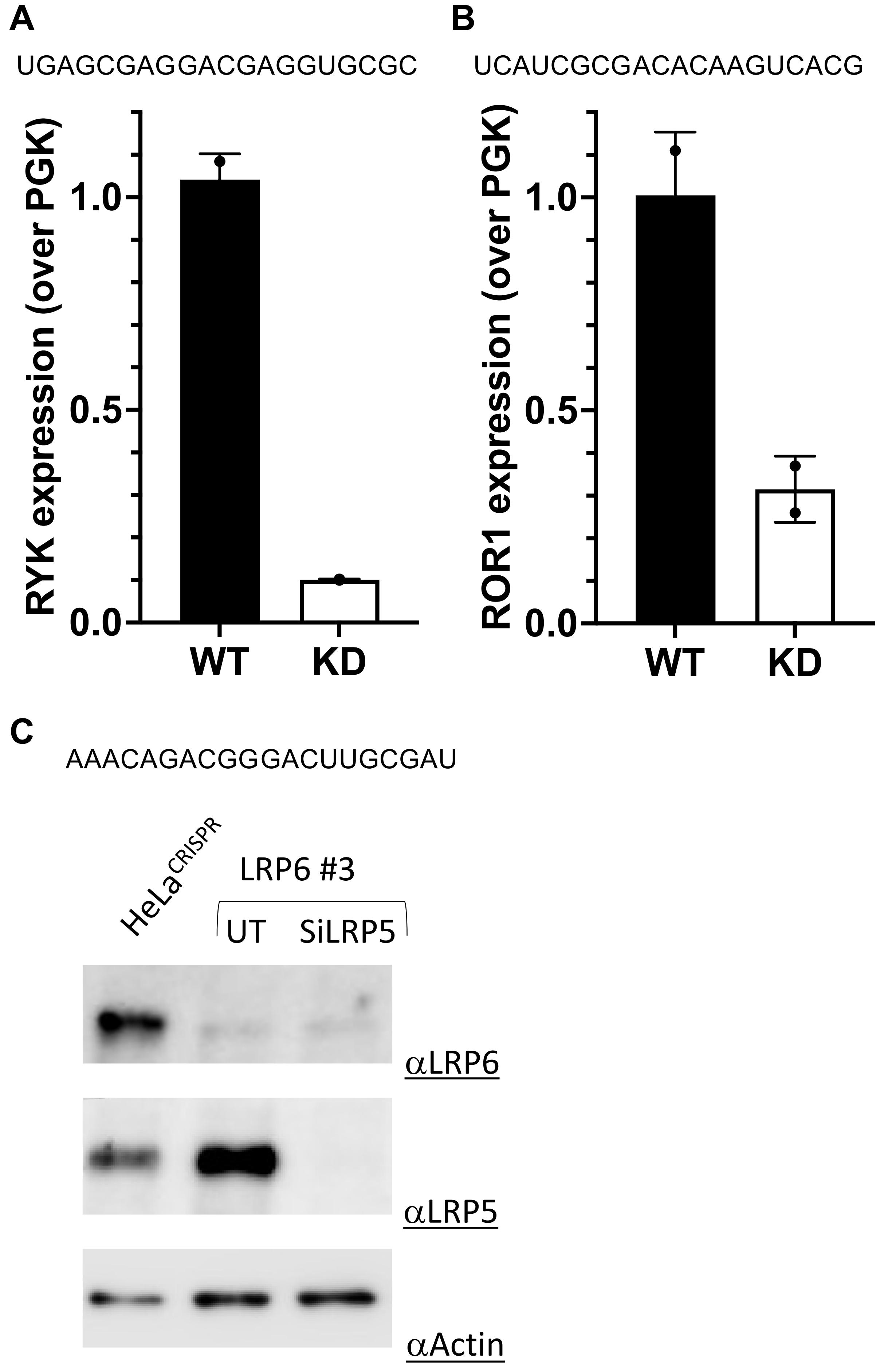

Figure S6 – Knockdown cell lines characterization

A. qPCR was performed on HeLa^{CRISPR} and a clonal RYK knockdown cell line to test for RYK expression. The sgRNA sequence used to target RYK is indicated. B. qPCR was performed on HeLa^{CRISPR} and a clonal ROR1 knockdown cell line to test for ROR1 expression. The sgRNA sequence used to target ROR1 is indicated. C. Western blot was performed on HeLa^{CRISPR}, and a clonal LRP6 knockdown cell line that was left untreated (UT) or further transfected with SiRNA targeting LRP5. Actin was used as a loading control. The sgRNA sequence used to target LRP6 is indicated.

Figure S7. EV binding assay

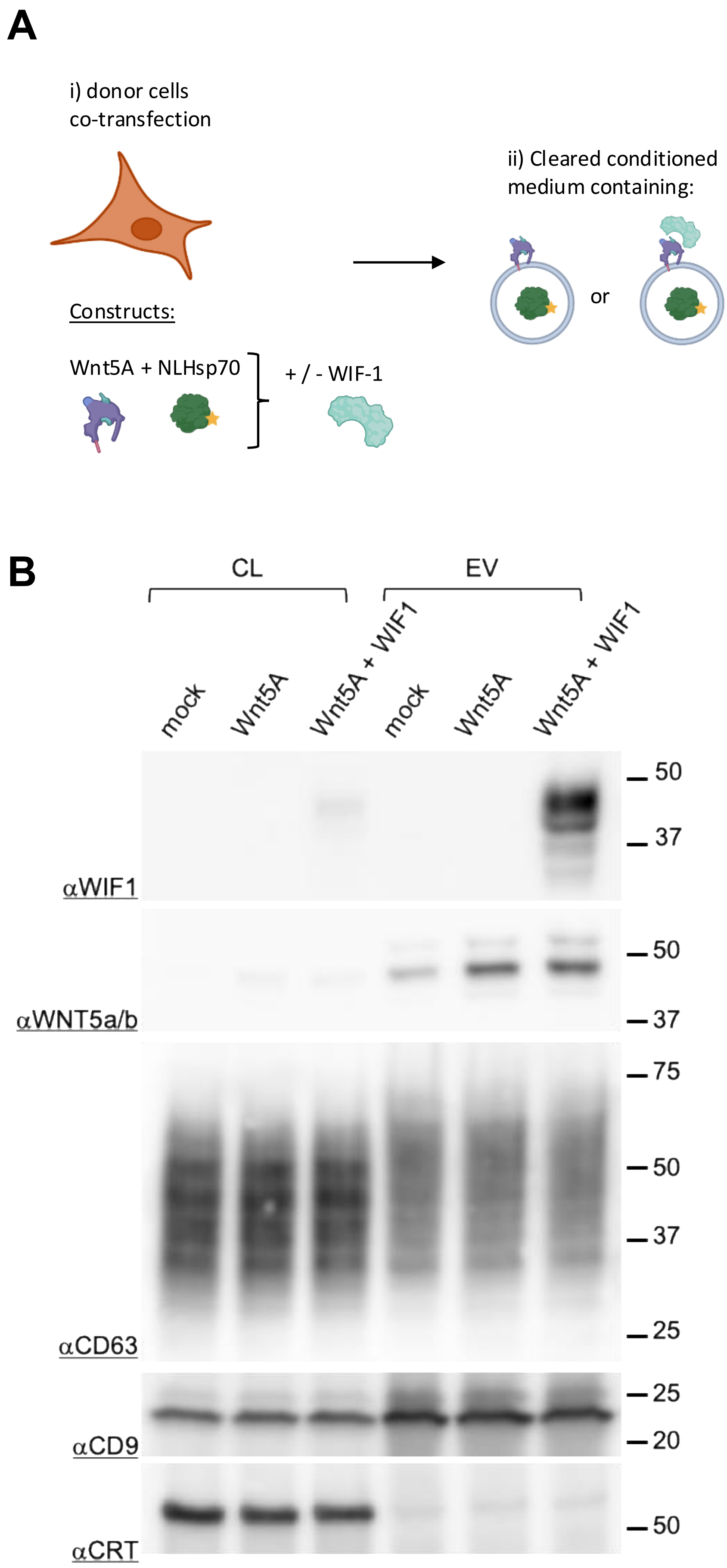

Figure S7 – EV binding assay

A. Scheme of the EV binding assay: donor cells were transfected with NLHsp70 as an EV tracer along with Wnt5A with or without WIF1. EV-containing conditioned medium from donor cells was cleared from cell debris and incubated with acceptor cells (either WT or RYK KO) on ice to inhibit internalization. After washing, cell-associated luminescence was measured. Created in BioRender. Dancourt, J. (2026) xnngj9u B. Equivalent amounts of proteins from HeLa donor cell lysates (CL) transfected with the indicated plasmids, or EVs produced from these cells were subjected to western blotting with antibodies directed against the indicated proteins. CRT: calreticulin.

Figure S8. Wnt pathways differentially influence EV uptake

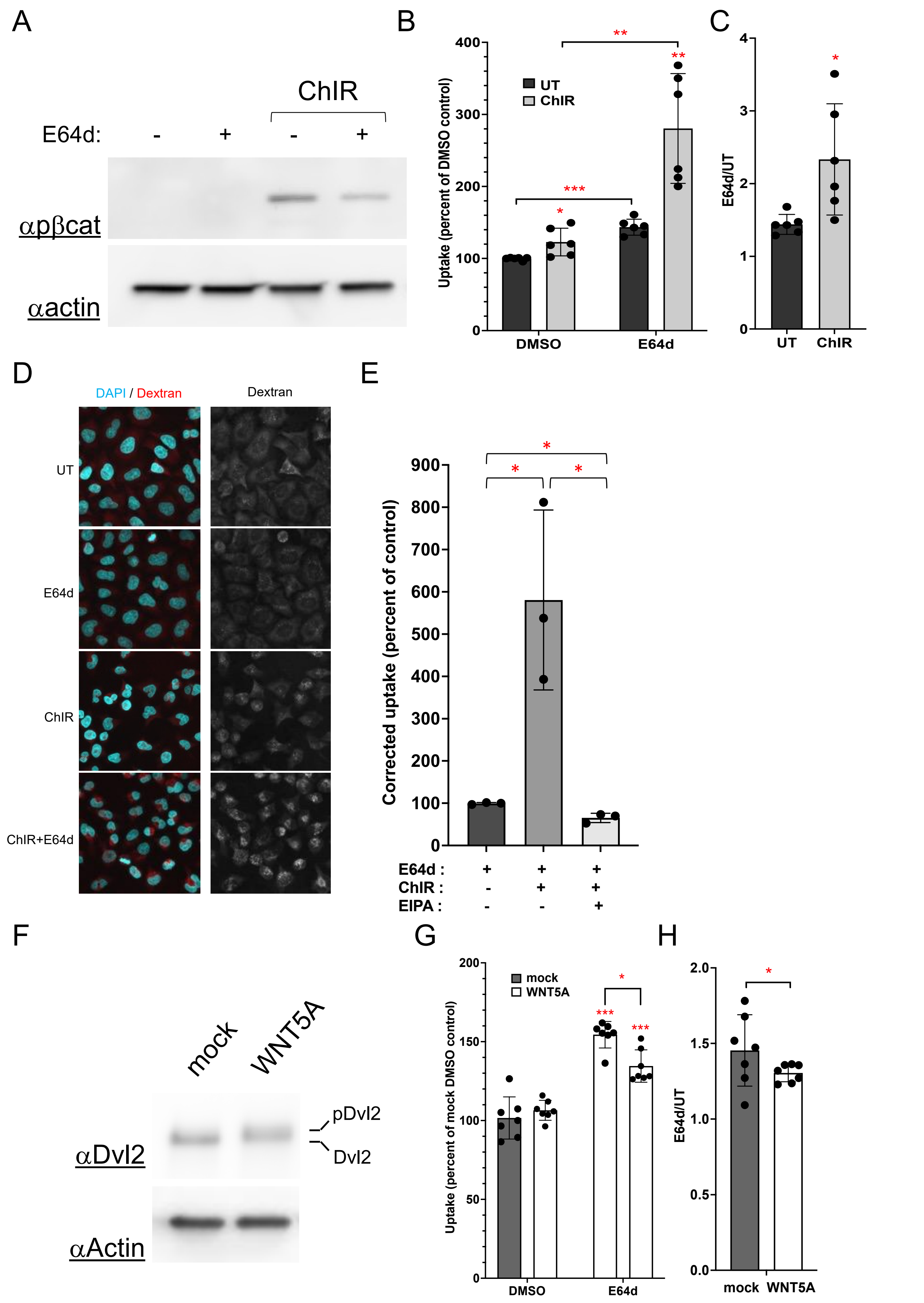

Figure S8 –Wnt pathways differentially influence EV uptake

A. Total proteins from cells treated with 6 μM ChIR99021 and/or 20 μM E64d or DMSO (-) were subjected to western blots with the indicated antibodies. B. EV uptake experiments were performed for 24 hours in the presence of 6 μM ChIR99021 or DMSO vehicle (untreated, UT) with or without 20 μM E64d (left panel). C. The E64d/UT ratio represents the EV uptake values from B in the presence of E64d divided by their DMSO control counterparts. D. A fluid phase marker (Lysine-fixable Texas Red 70 kDa dextran) was incubated with HeLa cells in the presence of 6 μM ChIR99021, 20 μM E64d, both or DMSO control for 2 hours before being fixed, labeled with DAPI and imaged. Dextran signal was increase in cells treated with ChIR99021 and this effect was potentiated with E64d. E. EV uptake was performed for 24 hours in the presence of 20 μM E64d with or without 6 μM ChIR99021 and/or 40 μM of the macropinocytosis inhibitor EIPA as indicated. EIPA abolished the effect of ChIR99021 on EV uptake. F. Total proteins from cells transfected with WNT5A or a mock plasmid were subjected to western blots with the indicated antibodies. G. EV uptake experiments were performed for 24 hours on recipient cells transfected with WNT5A or with a mock plasmid, with or without 20 μM E64d (left panel). H. The E64d/UT ratio represents the EV uptake values from G in the presence of E64d divided by their DMSO control counterparts. *: p<0,1, **: p<0,01, ***: p<0,001.

Figure S9. The amount of WNT-decorated EVs used in the uptake studies do not trigger detectable signaling

A

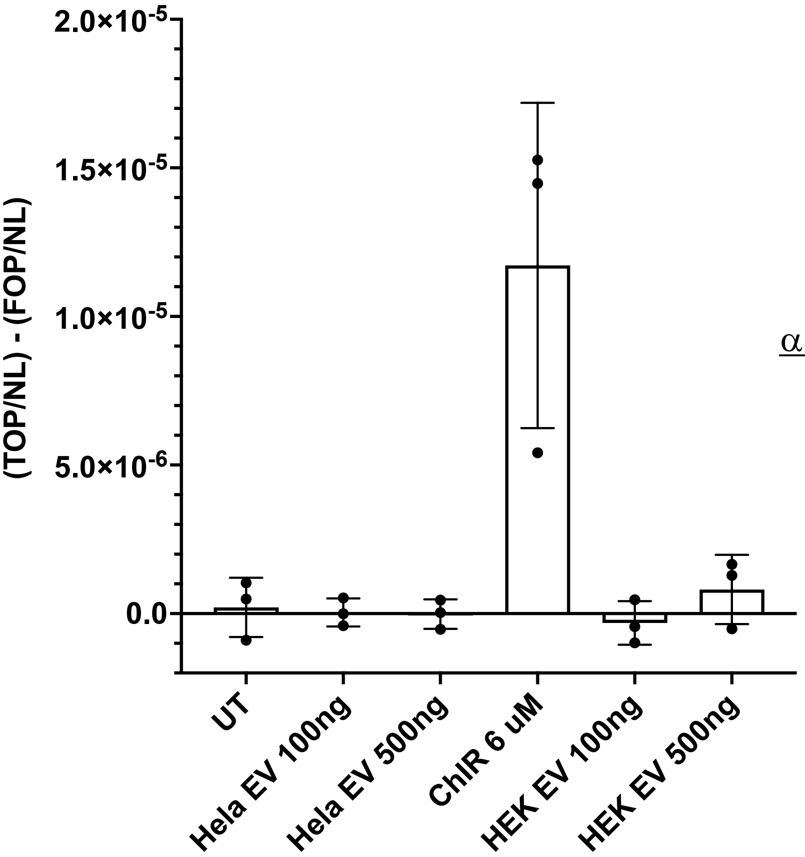

B

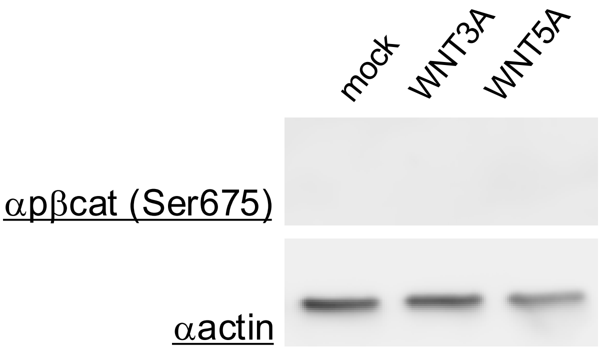

C

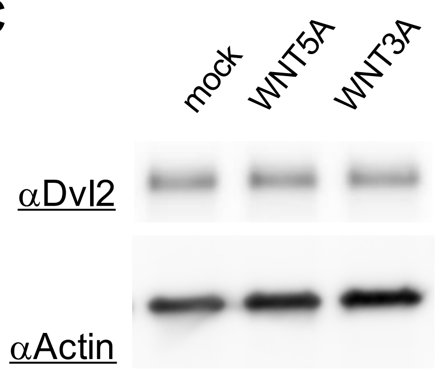

Figure S9 - The amount of WNT-decorated EVs used in uptake studies do not trigger detectable signaling

A. TOP/FOP assay to measure βcatenin activity in cells incubated with the indicated amount of EVs isolated from either HEK293 or HeLa cells. ChIR99021- and un-treated cells were respectively used as positive and negative control. For typical EV uptake experiment, 100ng of EVs were used. Note that no other condition than the positive control trigger any βcatenin signaling. B. Western blot of total proteins extracted from HeLa recipient cells incubated in the same conditions as for EV uptake assays with the indicated EV populations. Phosphorylation of either βcatenin (B) or Dvl2 (C) was assessed, along with actin expression used as a loading control. Note that βcatenin or Dvl2 phosphorylation was detected in none of the conditions tested.

Table S1. Plasmids used in this study

Construct expressed	Plasmid name	Reference
Cas9	lentiCRISPRv2 neo	Addgene, Cat #98292
NLCD63	JDB45	Bonsergent et al. (10)
NLHsp70	JDB2	Bonsergent et al. (10)
Empty vector	pCDNA3.1(-), mock	Invitrogen, Cat # V79020
WNT3A	pcDNA-Wnt3A	Addgene, Cat #35908
WNT5A	pcDNA-Wnt5A	Addgene, Cat #35911
WNT5A-V5	pcDNA3.2/V5-DEST-Wnt5A-V5	Addgene, Cat #43813
WIF-1	pcDNA3.1(-)-WIF1	Addgene, Cat #99533
GFPHSP70	pEGFP hsp70	Addgene, Cat #15215
sgRNA (puromycin^{R})	pGL3-U6-sgRNA-PGK-puromycin	Addgene, Cat #51133
RFPCD63	JDB43	Bonsergent et al. (10)
RFPRab5	RFPRab5	Kind gift, L. Johannes
TOPFlash	M50 Super 8x TOPFlash	Addgene, Cat #12456
FOPFlash mutant	M51 Super 8x FOPFlash mutant	Addgene, Cat #12457

Extended Data 1. Curated genetic screening results
